# Tissue specific requirement of *Drosophila* Rcd4 for centriole duplication and ciliogenesis

**DOI:** 10.1083/jcb.201912154

**Published:** 2020-06-16

**Authors:** Pallavi Panda, Levente Kovacs, Nikola Dzhindzhev, Agnieszka Fatalska, Veronica Persico, Marco Geymonat, Maria Giovanna Riparbelli, Giuliano Callaini, David M. Glover

**Affiliations:** 1 Department of Genetics, University of Cambridge, Cambridge, UK; 2 Institute of Biochemistry and Biophysics, Polish Academy of Science, Warsaw, Poland; 3 Division of Biology and Biological Engineering, California Institute of Technology, Pasadena, CA; 4 Department of Life Sciences, University of Siena, Siena, Italy

## Abstract

Rcd4 is a poorly characterized *Drosophila* centriole component whose mammalian counterpart, PPP1R35, is suggested to function in centriole elongation and conversion to centrosomes. Here, we show that *rcd4* mutants exhibit fewer centrioles, aberrant mitoses, and reduced basal bodies in sensory organs. Rcd4 interacts with the C-terminal part of Ana3, which loads onto the procentriole during interphase, ahead of Rcd4 and before mitosis. Accordingly, depletion of Ana3 prevents Rcd4 recruitment but not vice versa. We find that neither Ana3 nor Rcd4 participates directly in the mitotic conversion of centrioles to centrosomes, but both are required to load Ana1, which is essential for such conversion. Whereas *ana3* mutants are male sterile, reflecting a requirement for Ana3 for centriole development in the male germ line, *rcd4* mutants are fertile and have male germ line centrioles of normal length. Thus, Rcd4 is essential in somatic cells but is not absolutely required in spermatogenesis, indicating tissue-specific roles in centriole and basal body formation.

## Teaser

Panda et al. find that Rcd4 is required for centriole duplication and ciliogenesis in neurosensory organs but dispensable for spermatogenesis, indicating tissue specific requirements for centriole formation. Loading of Rcd4 and its binding partner Ana3 onto daughter centrioles is a prerequisite for the mitotic conversion of centrioles into centrosomes.

## Introduction

Centrioles are the core components of centrosomes and transform into the basal bodies of cilia. Consequently, defects in their structure, function, and duplication have an impact upon many aspects of biology ranging from cell division to cellular and developmental processes that require motility and signaling. Centriole dysfunction is associated with oncogenic transformation, inherited microcephaly, and a large variety of other genetic diseases with overlapping phenotypes collectively known as ciliopathies (Nigg and Raff, 2009; Bettencourt-Dias et al., 2011; Conduit et al., 2015).

To ensure that each cell has a single centrosome at each spindle pole during cell division, it is essential that the centriole replication process generates only a single copy of each centriole in each cell cycle. Each newly born G1 cell inherits a pair of centrioles, each of which becomes able to serve as a platform for the generation of a single new procentriole following their disengagement. The key players that regulate centriole duplication per se were discovered through genetic studies in *Caenorhabditis* *elegans* (O’Connell et al., 2001; Leidel and Gönczy, 2003; Kirkham et al., 2003; Dammermann et al., 2004; Delattre et al., 2004; Leidel et al., 2005; Kemp et al., 2004; Pelletier et al., 2004). These identified a protein kinase encoded by zygote defective 1 that acts upstream of the spindle assembly abnormal protein (Sas) 5 and Sas6, which are in turn upstream of Sas4. Zygote defective 1 protein is the *C. elegans* counterpart of the conserved polo-like kinase 4 (Plk4), master regulator of centriole duplication (Bettencourt-Dias et al., 2005; Habedanck et al., 2005); Sas5 corresponds to anastral spindle (Ana) 2 in flies and SCL/TAL1 interrupting locus (STIL) in humans (Arquint et al., 2015; Kitagawa et al., 2011; Kratz et al., 2015; Lettman et al., 2013; Moyer et al., 2015; Qiao et al., 2012; Stevens et al., 2010; van Breugel et al., 2011); Sas4 is also known as Sas4 in flies and as centrosomal P4.1-associated protein in humans (Basto et al., 2006; Kleylein-Sohn et al., 2007; Hsu et al., 2008). The phosphorylation of Ana2/STIL by Plk4 regulates its binding to Sas6 and their recruitment to a single site of procentriole formation (Dzhindzhev et al., 2014, 2017; Ohta et al., 2014). Excessive Plk4 activity drives multiple centriole formation, which is normally prevented through SKP1-CUL1-F-box-protein–mediated destruction of the kinase targeted at an auto-catalytically phosphorylated degron (Cunha-Ferreira et al., 2009, 2013; Klebba et al., 2013; Rogers et al., 2009). Nevertheless, the details of how procentriole formation is restricted to a single site remain to be uncovered. The subsequent recruitment of Sas4/centrosomal P4.1-associated protein promotes polymerization of centriolar microtubules that grow until capped by centriolar coiled coil protein 110 (Cp110) and its partner proteins (Hsu et al., 2008; Cottee et al., 2013; Hatzopoulos et al., 2013; Pelletier et al., 2006; Tang et al., 2011).

*Drosophila melanogaster Ana2* was one of three genes identified through an anastral spindle phenotype in a genome-wide RNA interference screen in cultured *Drosophila* cells (Goshima et al., 2007). The Ana1 protein has a human counterpart, centrosomal protein (Cep) 295, which was shown to be required for centriole to centrosome conversion, the final stage of the duplication cycle when daughter centrioles become competent to organize peri-centriolar material (PCM; Izquierdo et al., 2014). Without Cep295, new centrioles are destabilized when they lose their central cartwheel as daughters are converted into mothers. Ana1 has similar functions in cultured *Drosophila* cells, where it is recruited onto centrioles by Cep135 late in interphase and then serves as a means of recruiting Asterless (Asl; Fu et al., 2016). Asl is required to load Plk4 onto centrioles and also participates in recruiting PCM (Dzhindzhev et al., 2010; Novak et al., 2014; Conduit et al., 2014). In addition, the dosage of Ana1 influences the extent of centriole elongation (Saurya et al., 2016; Chang et al., 2016). Thus, Ana1 appears to have multiple functions. Studies of mutants for the third gene, *ana3*¸ suggest that it is required for the integrity of centrioles and basal bodies and centriole cohesion rather than centriole duplication per se (Stevens et al., 2009). Indeed, its human counterpart, Rotatin, directly interacts with STIL and has been shown to be required for the recruitment of the distal-half centriolar proteins, proteome of centriole 5 and proteome of centriole 1B. This appears to be mediated through a requirement for Rotatin upstream of CEP295 (Chen et al., 2017). These findings suggest Rotatin/Ana3 may have a role in either or both centriole to centrosome conversion and the assembly of the elongated centriole.

Another genome-wide screen confirmed the identity of many *Drosophila* genes required for centriole/centrosome functions and identified some new participants in these roles (Dobbelaere et al., 2008). Among the newly identified genes were several whose knockdown led to a reduced intensity of staining for the PCM component centrosomin. Of these, the Reduction in centrosomin dots 4 (*Rcd4*) gene product localized to centrosomes and was found to be required to maintain centrosome number in cultured cells. The roles of Rcd4 have never been examined in flies, and there is no knowledge of how it might fit into the hierarchies of centriole duplication, centriole to centrosome conversion, and/or centriole elongation. To address these questions, we now describe the generation and characterization of *Drosophila rcd4* mutants. The phenotypes of such mutants identify roles for Rcd4 in organizing the structure of centrioles and basal bodies. We show that Rcd4 and Ana3 directly interact to form a heterodimeric complex and that the timing of its recruitment to the centriole suggests a role in centriole to centrosome conversion.

## Results

### *rcd4 *mutant flies display defects characteristic of centriole loss

**Figure 1.**
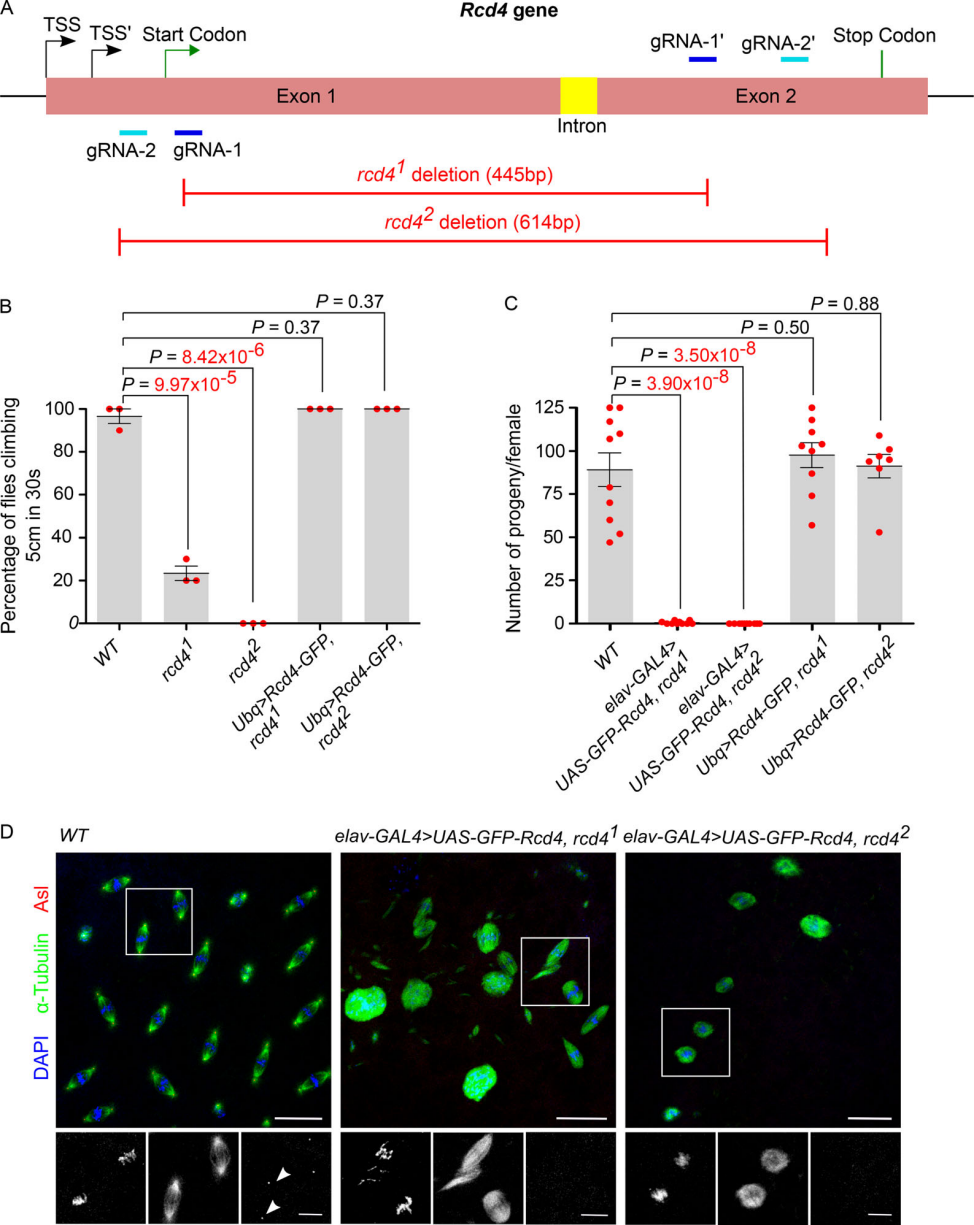
***rcd4* mutant flies are uncoordinated and female sterile due to centrosome loss. (A)** Schematic of CRISPR/Cas9-targeted mutagenesis of *Rcd4*, indicating the gRNA pair binding sites (light and dark blue) and the resultant deletion alleles (red). *Rcd4* has two alternative transcription start sites (TSS). **(B)** Climbing ability (coordination) of WT, *rcd4^1^*, *rcd4^2^*, and rescued flies (C-terminal GFP-tagged Rcd4 expressed under *Ubq* promoter in a *rcd4* mutant background). Cohorts of *n* = 10 flies were scored for each genotype for the percentage of flies that could climb 5 cm in 30 s. Means ± SEM are shown for three independent experiments. P values of two-tailed unpaired *t* tests are shown and values with significant difference are in red (99% confidence level). **(C)** Female fertility test of WT, coordination-rescued *rcd4^1^* and *rcd4^2^*, and rescued flies as in B. Females of each genotype were individually mated with WT males. Data points represent number of progeny from individual females. Means ± SEM are shown for at least *n* = 7 females per genotype. P values of two tailed unpaired *t* tests are shown, and values with significant difference are in red (99% confidence level). **(D)** Embryos from WT, coordination-rescued *rcd4^1^*, and *rcd4^2^* females as in C were immuno-stained to reveal Asl (red), α-tubulin (green), and DNA (blue). *n* = 20–30 embryos were analyzed per genotype, and all displayed the indicated phenotype; each experiment was repeated (N) at least two times. Arrowheads, centrosomes at spindle poles. Scale bars, 25 µm and 10 µm (inset).

Previous findings that GFP-tagged Rcd4 localizes to centrioles in cultured *Drosophila* cells and that its depletion leads to centrosome loss and a reduction in PCM recruitment (Dobbelaere et al., 2008) strongly suggest a role for the protein in the centriole duplication cycle. However, as the function of Rcd4 had never been examined in a whole organism, we turned to CRISPR/Cas9 mutagenesis to generate *rcd4* mutant flies (see Materials and methods). We generated two mutant alleles: *rcd4^1^* (NT_033779.5:g. 8382966_8383410del), a deletion allele with an intact start codon encoding a gene product lacking amino acids 4 to 131 but with an in-frame 68 amino acids C-terminal end; and *rcd4^2^* (NT_033779.5:g. 8382933_8383546del), a predicted null allele in which the start codon and a substantial portion of the coding sequence are deleted (Fig. 1 A).

Homozygous *rcd4^1^* and *rcd4^2^* flies showed lethality at the late pupal and adult stages (Table 1). The flies that emerged were slow and uncoordinated, a phenotype that was more severe in *rcd4^2^* flies, which were entirely unable to move about (Table 1; Fig. 1 B). As it has been previously reported that coordination defects in flies are a consequence of defective type I neurosensory cilia (Dubruille et al., 2002), we therefore asked whether the coordination defect could be rescued with a pan-neural driver *elav-GAL4* and the WT transgene upstream activating promotor**(*UAS*)*-GFP-Rcd4.* Indeed, this restored the coordination of the mutant flies, confirming that this defect was a consequence of defective neurosensory cilia. It also allowed us to test the fertility of *rcd4^1^* and particularly *rcd4^2^* flies, which were otherwise too uncoordinated to be able to mate successfully. We found that *rcd4^2^* flies with restored coordination were male fertile but showed female sterility (Table 1; Fig. 1 C). The male fertility accorded with the presence of motile sperm that we observed in *rcd4^2^* males even in the absence of the WT transgene *UAS-GFP-Rcd4*. The *rcd4^1^* flies exhibited sufficient coordination, but both the lethality and loss of coordination became aggravated when heterozygous to the *rcd4^2^* allele or a deficiency for the region, indicating that *rcd4^1^* is a hypomorph (Table 1; Fig. 1, B and C). Both the uncoordination and female sterility phenotypes of *rcd4^1^* and *rcd4^2^* flies could be rescued by ubiquitously expressing a WT *Rcd4-GFP* transgene (Table 1; Fig. 1, B and C), indicating that the defects observed are a consequence of the mutations in *Rcd4*. The female sterility of both *rcd4^1^* and *rcd4^2^* is consistent with possible loss of centrioles, which are required for centrosome function in the rapid mitoses of the syncytial embryo.

**Table 1.** Primary phenotypes observed in *rcd4* mutant flies

	CyO	*rcd4^1^*	*rcd4^2^*	Df(2L) ED626	*Ubq>Rcd4-GFP, rcd4^(1 or 2)^*	*elav-GAL4> UAS-GFP-Rcd4, rcd4^(1 or 2)^*
***rcd4*^*1*^**	Viable	Some adult lethal	Adult lethal	Adult lethal	Viable	Viable
	Coordinated	Moderately uncoordinated	Uncoordinated	Uncoordinated	Coordinated	Coordinated
	Fertile	Female sterile	Female sterile	Female sterile	Fertile	Female sterile
***rcd4^2^***	Viable	Adult lethal	Pharate/adult lethal	Pharate/adult lethal	Viable	Viable
	Coordinated	Uncoordinated	Severely uncoordinated	Severely uncoordinated	Coordinated	Coordinated
	Fertile	Female sterile	Female sterile	Female sterile	Fertile	Female sterile

Summary of the complementation tests for viability, coordination, and fertility. CyO*,* balancer second chromosome; Df(2L) ED626, deficiency allele covering *Rcd4*; *Ubq*, *polyubiquitin* promoter; *elav-GAL4,* pan-neuronal *GAL4* driver.

To determine whether the female sterility of *rcd4* mutant mothers did indeed reflect maternal effect lethality resulting from loss of centrosomes, we examined the nuclear division cycles of *rcd4* mutant–derived embryos. In WT syncytial embryos, there are 13 rapid rounds of synchronous nuclear division cycles, which rely upon a dowry of maternally provided cell cycle proteins. Throughout these division cycles, centrosomes are associated with the interphase nuclei and the poles of the mitotic spindles (Fig. 1 D). However, embryos derived from *rcd4^1^* mothers exhibited extensive disorganization of the mitotic apparatus from very early stages of development. Typically, the spindles had a collapsed appearance with dispersed microtubules, and we could detect negligible, if any, centrosomes at the spindle poles by staining for Asl (Fig. 1 D). We observed similar, but more severe, disorganization of the mitotic apparatus in embryos derived from *rcd4^2^* mothers. Thus, in accord with observations in cultured cells (Dobbelaere et al., 2008), the Rcd4 protein appears to be required for the multiple cycles of centriole duplication that drive the centrosome cycle in the syncytial *Drosophila* embryo.

**Figure S1.**
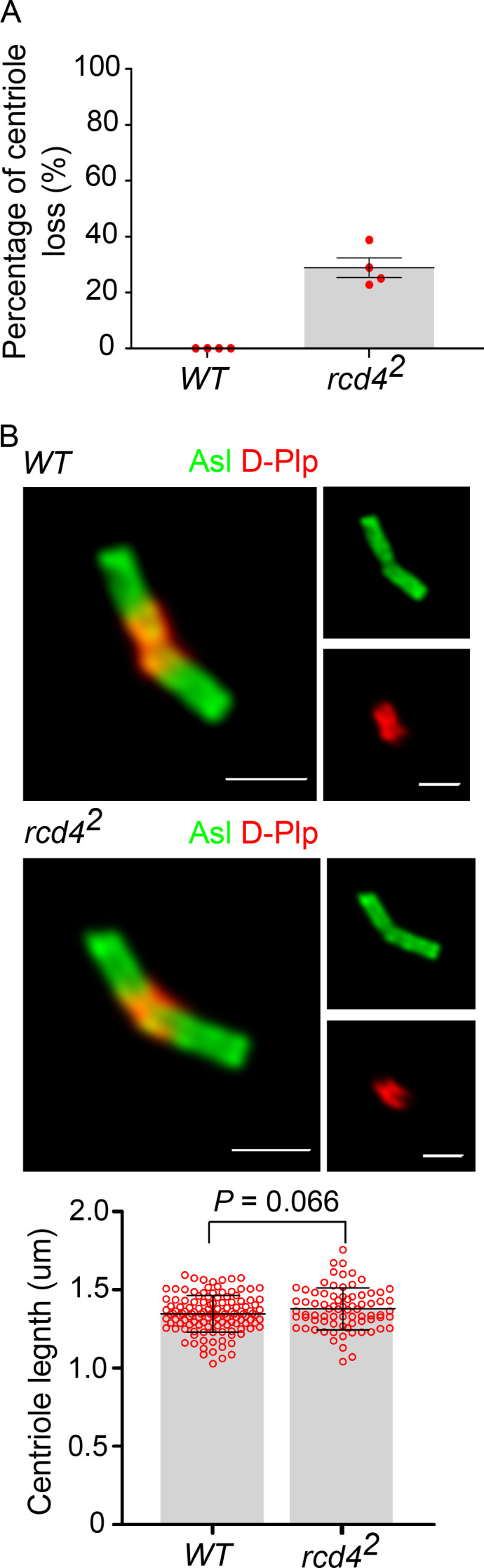
**Centriole elongation proceeds normally in *rcd4-*null mutant male germ line despite 29% centriole loss in primary spermatocytes. (A)** Percentage of centriole loss in WT and *rcd4^2^* primary spermatocytes. Mean ± SEM is shown for four testes for each genotype; *n* = 18–23 primary spermatocytes were analyzed in each testis; N = two times. **(B)** Representative images of giant centrioles from mature primary spermatocytes of WT and *rcd4^2^* flies immunostained to reveal pan-centriolar marker Asl (green) and D-Plp (red). Graph shows centriole length quantification using Asl as a marker. Mean ± SEM is shown for at least *n* = 75 centrioles measured from a total of five testes for each genotype; N = at least three times. P value of two-tailed unpaired *t* test is shown (99% confidence level). Scale bar, 1 µm.

In contrast to the maternal effect shown by *rcd4-*mutants, their male fertility suggested that centriole development should not be affected in the male germ line. Mature centrioles are generated in the primary spermatocytes, which undergo an extended growth phase in the G2 preceding meiosis, during which centrioles elongate to ∼1.3 µm. In *rcd4^1^* males, we found primary spermatocytes with two pairs of centrioles typical of those seen in WT spermatocytes. However, *rcd4^2^* primary spermatocytes showed around ∼29% of centriole loss (Fig. S1 A). Nevertheless, the *rcd4-*mutant centrioles had Asl along their entire length that was similar to WT centrioles (Fig. S1 B). Thus, although Rcd4 is essential for the development of centrioles within the somatic tissues of *Drosophila*, male fertility and limited centriole loss in *rcd4-null* mutant primary spermatocytes suggest that it is not absolutely required for the formation of the large centrioles of the male germ line.

### Coordination defects of *rcd4* mutant flies reflect failure of ciliary axoneme development in chordotonal organs

**Figure 2.**
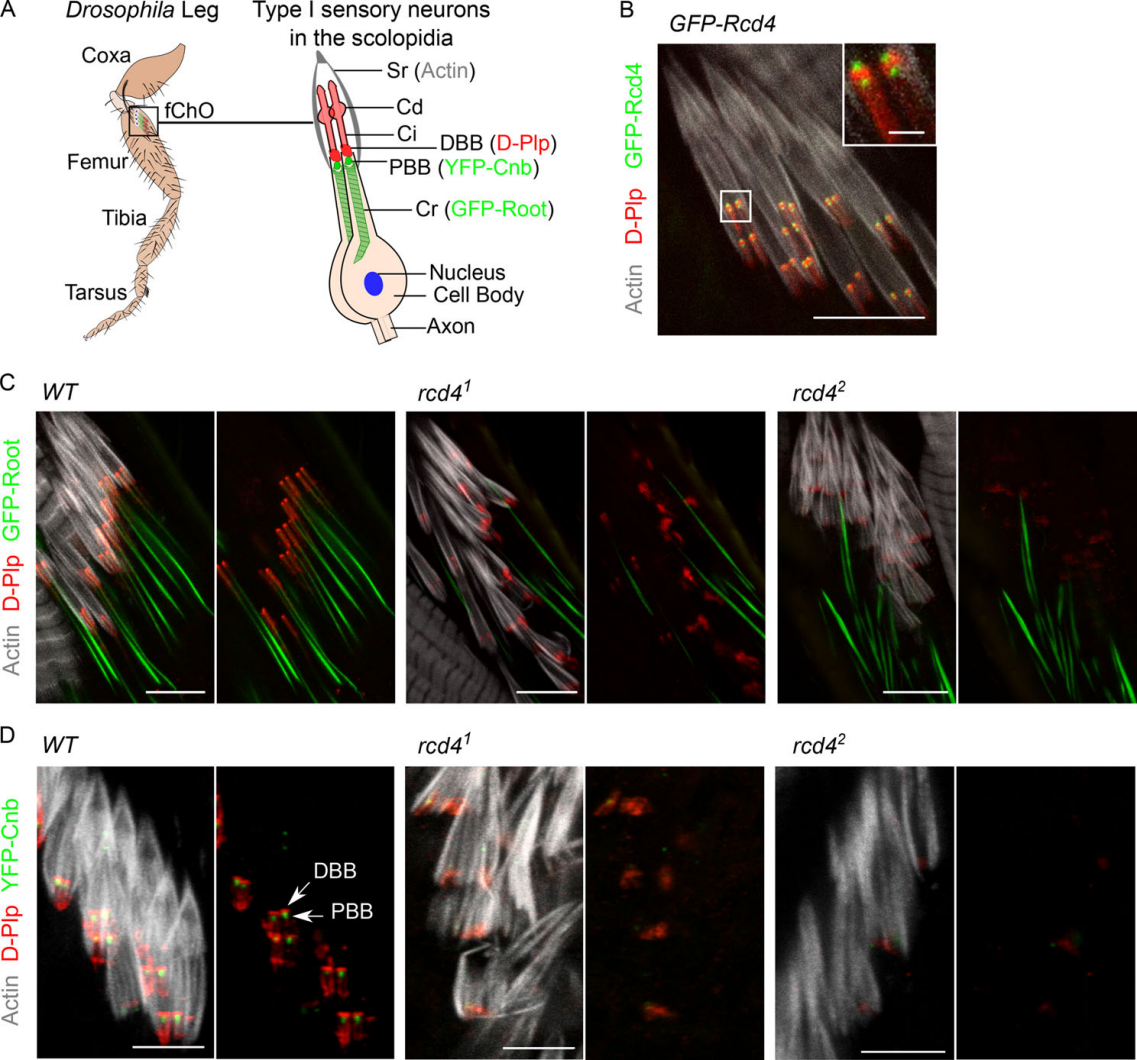
***rcd4* mutant flies show progressive loss of basal bodies in ciliated sensory neurons of the femoral chordotonal organ.** **(A)** Illustration of type I sensory neurons of *Drosophila* fChO. Cd, ciliary dilation; Ci, cilium; Cr, ciliary rootlet; Sr, scolopale rod. **(B)** GFP-Rcd4 (green) localization to basal bodies immunostained to reveal D-Plp (red) and actin (gray). Scale bars, 10 µm and 1 µm (inset). **(C)** WT and *rcd4* mutants expressing transgenic GFP-Rootletin (GFP-Root expressed under pan-neural driver *elav-GAL4* in a *rcd4* mutant background; green), immunostained as in B. *n* = 10 fly legs from five pharate adult flies were analyzed per genotype, and all displayed the indicated phenotype; N = at least three times. Scale bar, 10 µm. **(D)** WT and *rcd4* mutants expressing YFP-centrobin (YFP-Cnb; expressed under *Ubq* promoter in a *rcd4* mutant background; green), a daughter centriole (PBB) specific marker, immunostained as in B. *n* = 10 fly legs from five pharate adult flies were analyzed per genotype and all displayed the indicated phenotype; N = at least two times. Arrows, DBB and PBB. Scale bar, 5 µm.

We next wished to understand how the *rcd4* mutations resulted in the loss of coordination in mutant flies by examining the type-I ciliated sensory neurons of the femoral chordotonal organs (fChOs) of adult fly legs. To this end, we first examined the localization of Rcd4 within these organs. The chordotonal organs comprise clusters of scolopidia, within each of which are two ciliated neurons together with their accessory cells. Each neuron’s distal basal body (DBB), derived from the mother centriole, is responsible for templating the formation of the cilium. The proximal basal body (PBB), derived from the daughter centriole, is connected to the cell body by the ciliary rootlet (Fig. 2 A). We found that GFP-tagged Rcd4 was associated with the two basal bodies in each scolopidium in relation to centriolar protein *Drosophila* pericentrin-like protein (D-Plp), which showed a more diffused accumulation between each DBB and PBB at the base of the actin-enshrouded cilia (Fig. 2 B).

**Figure S2.**
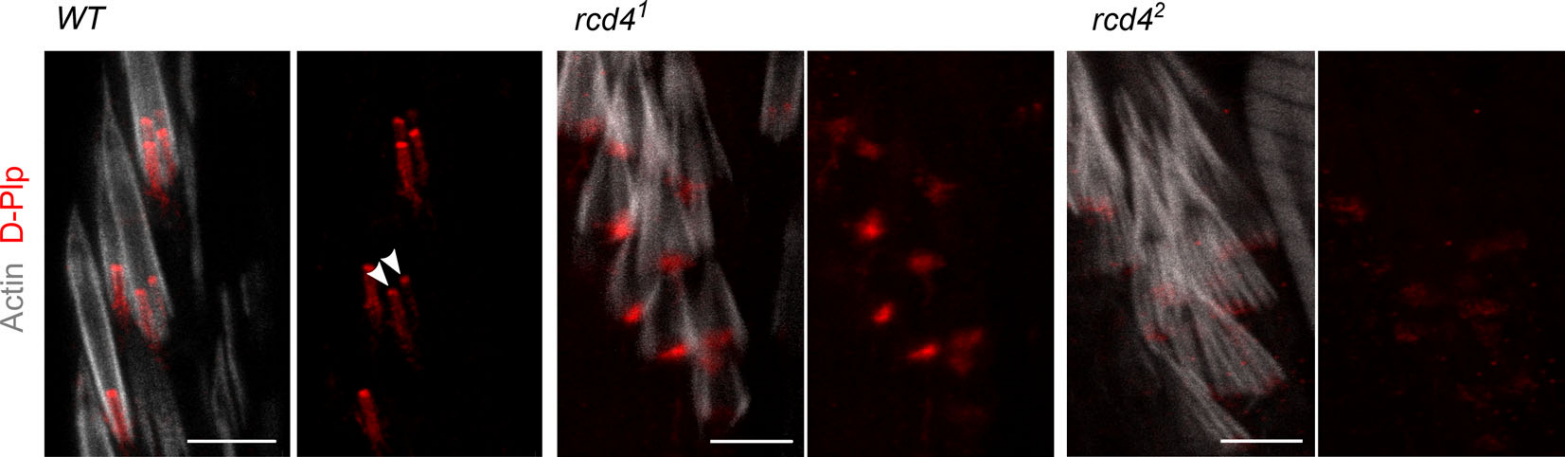
**Disrupted D-Plp staining is indicative of defective basal bodies in *rcd4-*mutant fChOs.** WT and *rcd4* mutant fChOs immuno-stained to reveal D-Plp (red) and actin (gray). *n* = 10 fly legs from five pharate adult flies per genotype; all fly legs displayed the indicated phenotype; N = at least three times. Arrowheads, individual basal bodies (DBBs). Scale bar, 5 µm.

We then examined the organization of fChOs in the *rcd4-*mutants. WT fChOs have an ordered array of scolopidia revealed by transgenic GFP-Rootletin staining of the ciliary rootlet, D-Plp staining of the basal bodies, and phalloidin staining of the actin bundles that envelop the cilia (Fig. 2 C). In contrast, we found that *rcd4^1^* fChOs were highly disorganized: ciliary rootlets were rarely observed in ordered pairs as in the WT, were missing in many of the scolopidia, or did not form junctions with basal bodies; D-Plp staining was reduced and diffused, suggesting defects or absence of basal bodies; and the actin bundles were disorganized (Fig. 2 C). These mutant phenotypes were more severe in the *rcd4^2^*. Closer inspection revealed that the majority of *rcd4^1^* scolopidia had only a single D-Plp body, suggesting that only a single cilium could be specified in such cells, whereas *rcd4^2^* scolopidia lacked a D-Plp signal (Fig. S2). The PBB derived from the daughter centriole can be distinguished from the one derived from the mother by its association with centrobin, a daughter centriole protein required for centriole duplication (Zou et al., 2005). However, the fChOs of neither *rcd4* mutant alleles had any centrobin-positive basal bodies, indicating a failure in centriole duplication (Fig. 2 D).

**Figure 3.**
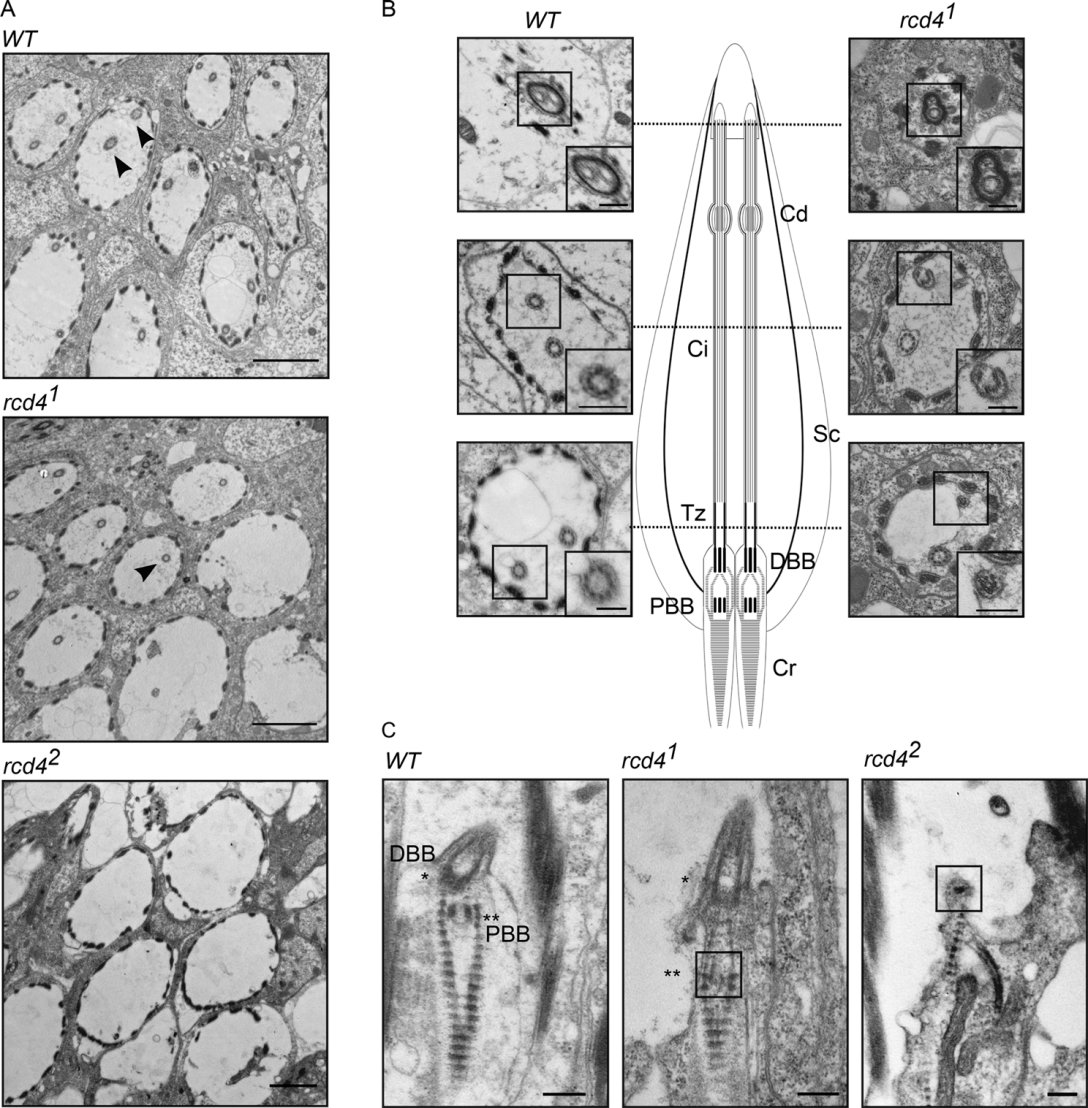
**Transmission electron microscopy reveals basal body organizational defects and loss of ciliary axoneme in *rcd4* mutant chordotonal organs (Johnston’s organ).** **(A)** Transverse sections of WT and *rcd4* mutant chordotonal organs across the ciliary region, *n* = 53, 180, and 128 scolopidia scored for WT, *rcd4^1^*, and *rcd4^2^*, respectively. N = at least three times. Arrowheads indicate the ciliary axoneme within each individual scolopidium. Scale bar, 2 µm. **(B)** Transverse sections through the transition zone, cilia, and above the ciliary dilation of WT and *rcd4^1^* scolopidia as illustrated in the diagram. Cd, ciliary dilation; Ci, cilium; Cr, ciliary rootlet; Sc, scolopale cell; Tz, transition zone. N= two times. Scale bar, 200 nm. **(C)** Longitudinal sections of the basal body region in WT and *rcd4* mutants. N = two times. Single asterisk, DBB; double asterisk, PBB; marked box, basal body remnants in *rcd4* mutants. Scale bar, 250 nm.

**Figure S3.**
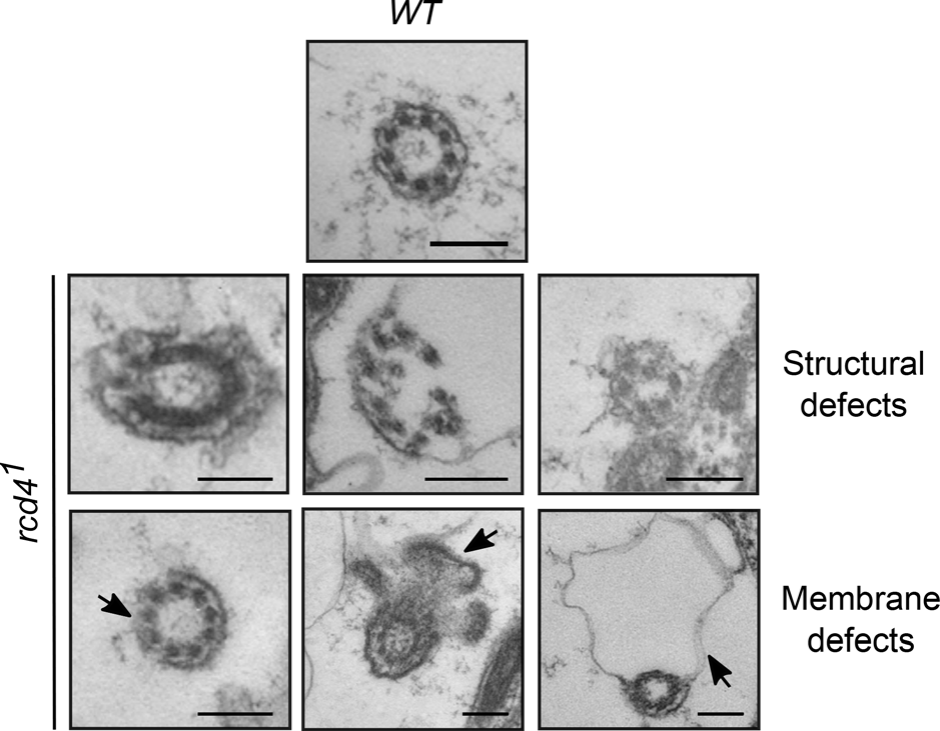
**Ciliary axoneme abnormalities observed in *rcd4^1^* chordotonal organ (Johnston's organ).** Transmission electron microscopy transverse sections of WT and *rcd4^1^* cilia. N = two times. Arrows, abnormal ciliary membrane organization. Scale bar, 200 nm.

The formation of the sensory cilia in the fChOs requires basal bodies that have no associated centrobin and are thus distal basal bodies corresponding to mother centrioles (Gottardo et al., 2015). As *rcd4^1^* scolopidia had D-Plp–containing bodies lacking centrobin, we anticipated that these may be able to template cilia. However, the absence of D-Plp staining in *rcd4^2^* suggested that it would be incapable of directing cilia formation. To determine whether this distribution of D-Plp truly reflected a reduced number of basal bodies and cilia in the *rcd4-*mutant fChOs, we turned to electron microscopy. Transverse sections of the WT antennal Johnston’s organ scolopidia revealed the expected pair of ciliary axonemes, whereas ∼40% of *rcd4^1^* scolopidia had just a single ciliary axoneme, and 57% had no axonemes at all (Fig. 3 A). Abnormalities were present throughout the length of the cilia. Transverse sections of the cilia proximal and distal to the ciliary dilation and of the transition zone revealed the expected two axonemes of ninefold symmetry extending throughout the length of WT scolopdia, whereas in the ∼3% of cases where *rcd4^1^* scolipidia had two ciliary axonemes, one usually had a WT appearance, whereas the other displayed abnormalities in symmetry and structural integrity (Fig. 3 B). When a single cilium was present in the *rcd4^1^* scolopidium, this could have a WT or structurally abnormal appearance in the transverse section and was often associated with defects in the organization of the ciliary membrane (Fig. S3). Longitudinal sections revealed the mother-daughter pair of basal bodies in WT scolopidia. However, *rcd4^1 ^*mutants were associated with a DBB of apparently normal architecture and a disorganized or absent PBB, while *rcd4*^*2*^-mutants were left with only remanents of basal bodies (Fig. 3 C). Together these observations point to progressive failure in the duplication of centrioles in the final cell divisions of the scolopidium cell lineage in the *rcd4^1^* mutant such that only a mother centriole remains. Consistent with the absence of centrobin from mother centrioles, these residual centrioles become basal bodies able to direct formation of cilia. However, the majority of such cilia are structurally defective.

Moreover, in agreement with the genetic findings (Fig. 1), failure of centriole duplication was more pronounced in *rcd4^2^*, where we could not detect any ciliated scolopidium in the Johnston’s organ of the antenna (Fig. 3 A) Thus, together our findings indicate that the loss of coordination in *rcd4* mutants reflects the abnormal anatomy of the fChOs and that this can be accounted for by a failure in the centriole duplication cycle.

### Rcd4 is a binding partner of Ana3

To assess the functions of Rcd4 within the centriole, we next wished to identify its interaction partner. We therefore affinity-purified GFP-tagged Rcd4 from cultured cells and analyzed the complexes by mass spectrometry. This identified a single known centriolar protein with high Mascot scores, namely Ana3, also shown to be essential for the duplication cycle (Table 2; Stevens et al., 2009). A reciprocal experiment with GFP-tagged Ana3 similarly revealed Rcd4 as the only other interacting centriolar protein (Table 2).

**Table 2.** Rcd4 and Ana3 copurify

D.Mel-2 cultured cells				
Proteins	pAct5C-Rcd4-GFP		pAct5C-Rcd4-GFP (+OA)	
	Score	Peptides	Score	Peptides
**Rcd4**	1001	30	1123	32
**Ana3**	5316	121	5212	110
	**pAct5C-GFP-Ana3**		**pAct5C-GFP-Ana3 (+OA)**	
**Ana3**	10438	332	13029	426
**Rcd4**	825	23	1042	32

Top: Copurification of Ana3 with Rcd4-GFP (bait) from cultured D.Mel-2 cells expressing Rcd4-GFP under the control of a constitutive actin-5c promoter. Bottom: Copurification of Rcd4 with GFP-Ana3 (bait) from cultured D.Mel-2 cells expressing GFP-Ana3 under the control of a constitutive actin-5 promoter. Extracts were made in isotonic buffer and with okadaic acid (OA) as indicated. Scores (mascot) and numbers of peptides detected by mass spectrometry are indicated.

**Figure 4.**
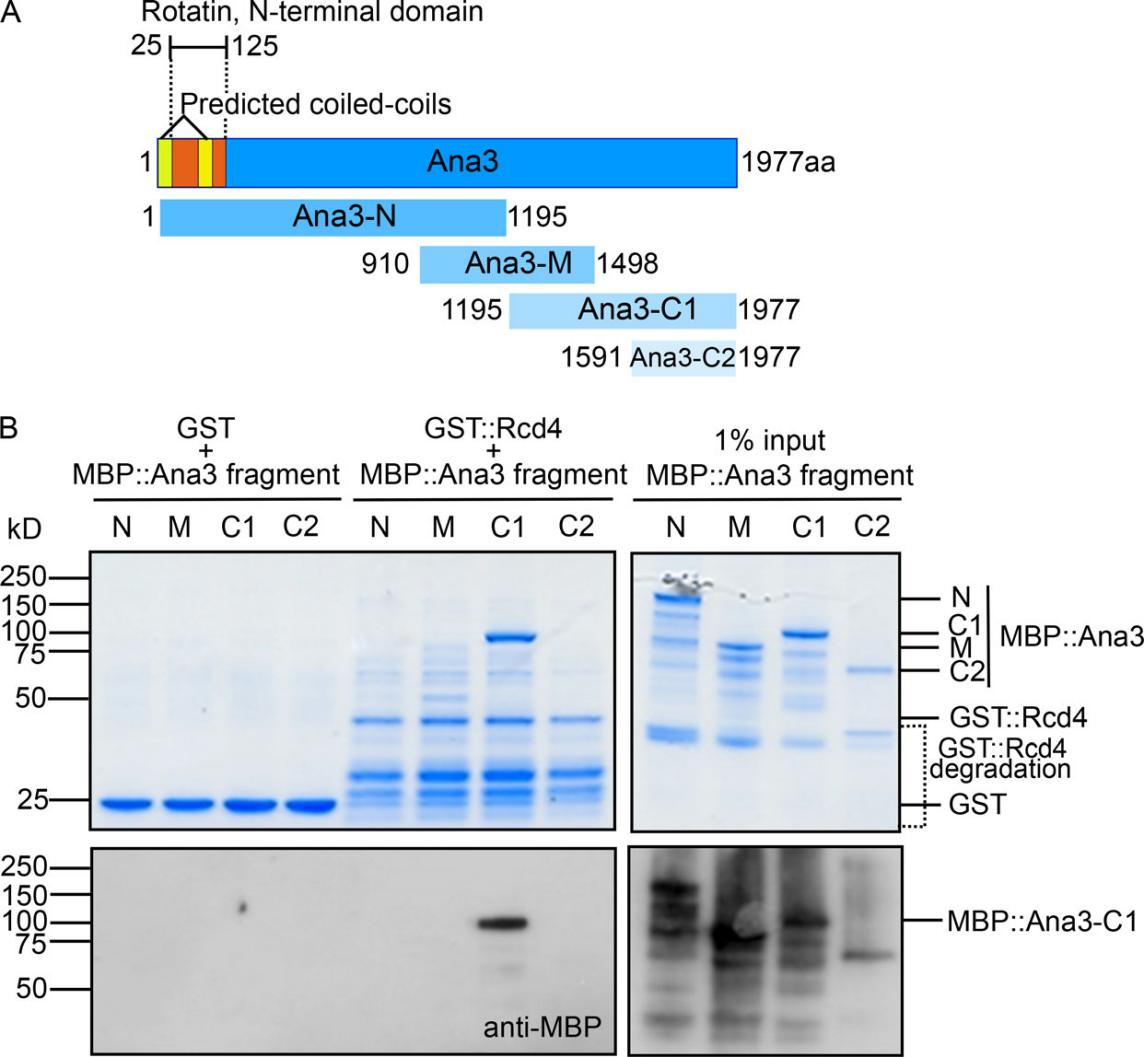
**Rcd4 directly interacts with Ana3. (A)** Schematic of Ana3 indicating predicted domains, structural annotations, and fragments used for binding studies. Ana3-N, Ana3-N terminal; Ana3-M, Ana3-middle; Ana3-C1, Ana3-C terminal 1; Ana3-C2, Ana3-C terminal 2. **(B)** In vitro binding assay between GST-tagged Rcd4 and MBP-tagged Ana3 fragments (as shown in A), both expressed and purified from *E. coli*. GST/GST::Rcd4 were purified, and the resulting complexes were analyzed by SDS-PAGE gel (Coomassie staining) and Western blotting to detect interactions with MBP::Ana3 fragments. N = at least three times.

To determine whether Rcd4 and Ana3 were direct binding partners, we assessed their interactions through in vitro binding studies. We expressed and purified overlapping fragments of the Ana3 protein from *Escherichia coli* (Fig. 4 A), each tagged with maltose binding protein (MBP). These were tested in vitro for their binding to Rcd4 tagged with GST, also produced and purified from *E. coli.* We purified the complexes on glutathione beads and eluted the protein for analysis by SDS-PAGE and Western blotting. This revealed strong binding between Rcd4 and the 1195–1977 amino acid (C1) segment of Ana3, confirming that Rcd4 and Ana3 could indeed form a complex in vitro (Fig. 4 B).

**Figure S4.**
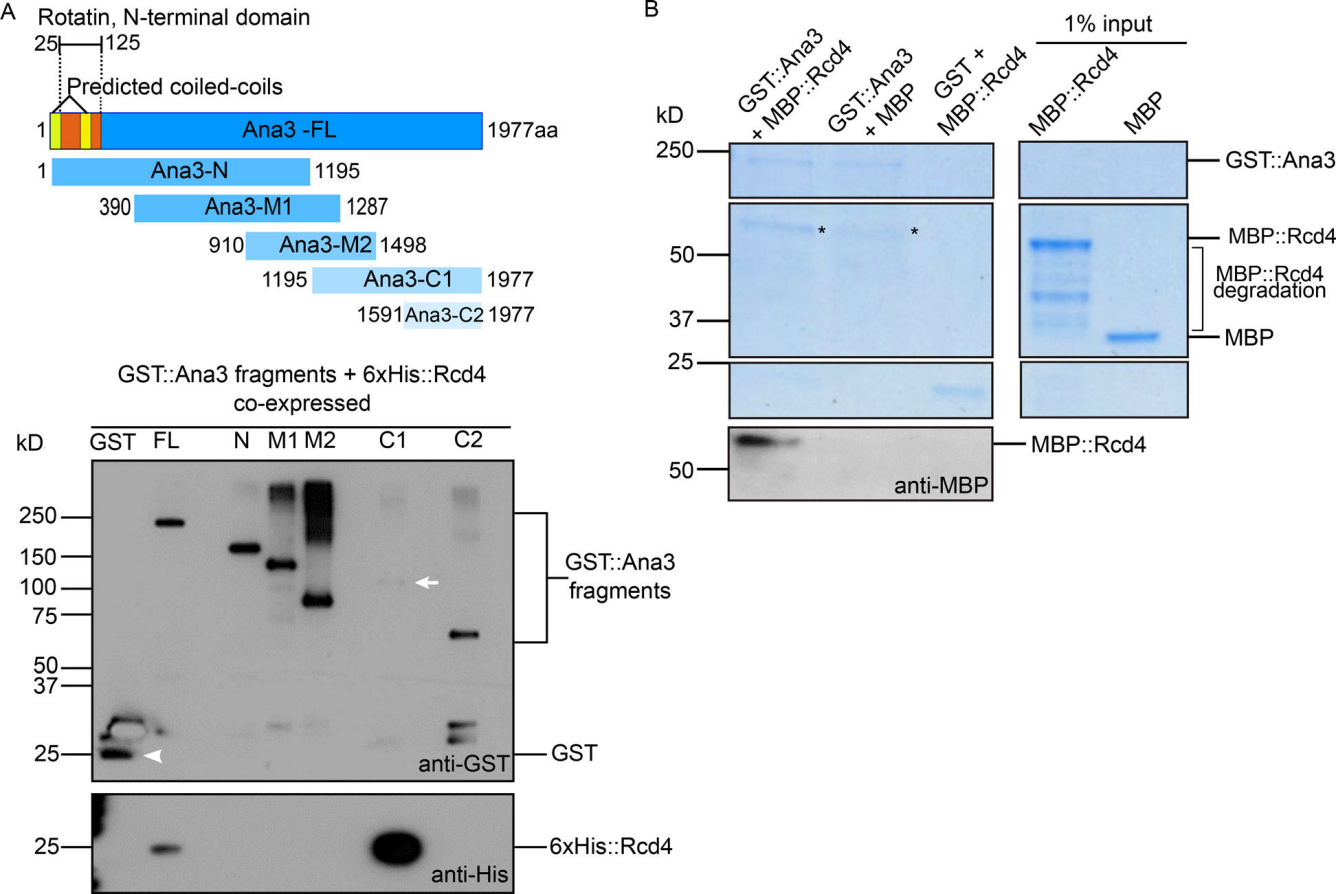
**Binding assays between full-length Ana3 and Rcd4.** **(A)** In vivo binding assay between GST-tagged Ana3 full-length/fragments (as depicted in the schematic; Ana3-FL, Ana3 full-length; Ana3-N, Ana3-N terminal; Ana3-M, Ana3-middle; Ana3-C1, Ana3-C terminal 1; Ana3-C2, Ana3-C terminal 2) and 6xHis-tagged Rcd4, coexpressed and purified from *S. cerevisiae*. GST::Ana3/GST were purified, and the resulting complexes were analyzed by Western blotting to detect interactions with 6xHis::Rcd4. N = two times. Arrowhead, GST; arrow, GST::Ana3 C1. **(B)** In vitro binding assay between full-length GST-tagged Ana3 and MBP-tagged Rcd4, expressed and purified from *S. cerevisiae* and *E. coli*, respectively. GST::Ana3/GST were purified, and the resulting complexes were analyzed by SDS-PAGE gel (Coomassie staining) and Western blotting to detect interactions with MBP::Rcd4. N = two times. Asterisk, background binding.

We repeatedly found that full-length Ana3 was recalcitrant to expression in bacterial cells and therefore sought to express the protein in *Saccharomyces cerevisiae*. We amplified the chemically synthesized full-length *Ana3*–coding sequence (CDS) or segments of it by PCR, and inserted these into a yeast expression plasmid by in vivo recombination such that the expressed proteins would be tagged with GST (Geymonat et al., 2007). We followed a similar strategy with 6xHis-tagged Rcd4 and coexpressed the GST- and 6xHis-tagged proteins in yeast for purification of the resulting complexes. We found that Rcd4 copurified with the GST-tagged full-length Ana3 and with the C-terminal 1195–1977 amino acid C1 fragment, confirming the in vitro binding experiment (Fig. S4 A).

Finally, we expressed and purified full-length GST-Ana3 from yeast and MBP-Rcd4 from bacterial cells, incubated these proteins together or with respective MBP or GST proteins alone, and assessed complex formation by SDS-PAGE and Western blotting. The results once again confirmed the complex formation between Rcd4 and Ana3 (Fig. S4 B). Taken together, these experiments demonstrate a direct interaction between Rcd4 and Ana3 in its C-terminal part.

### Rcd4 and Ana3 localize to the distal part of centriole zone I

**Figure 5.**
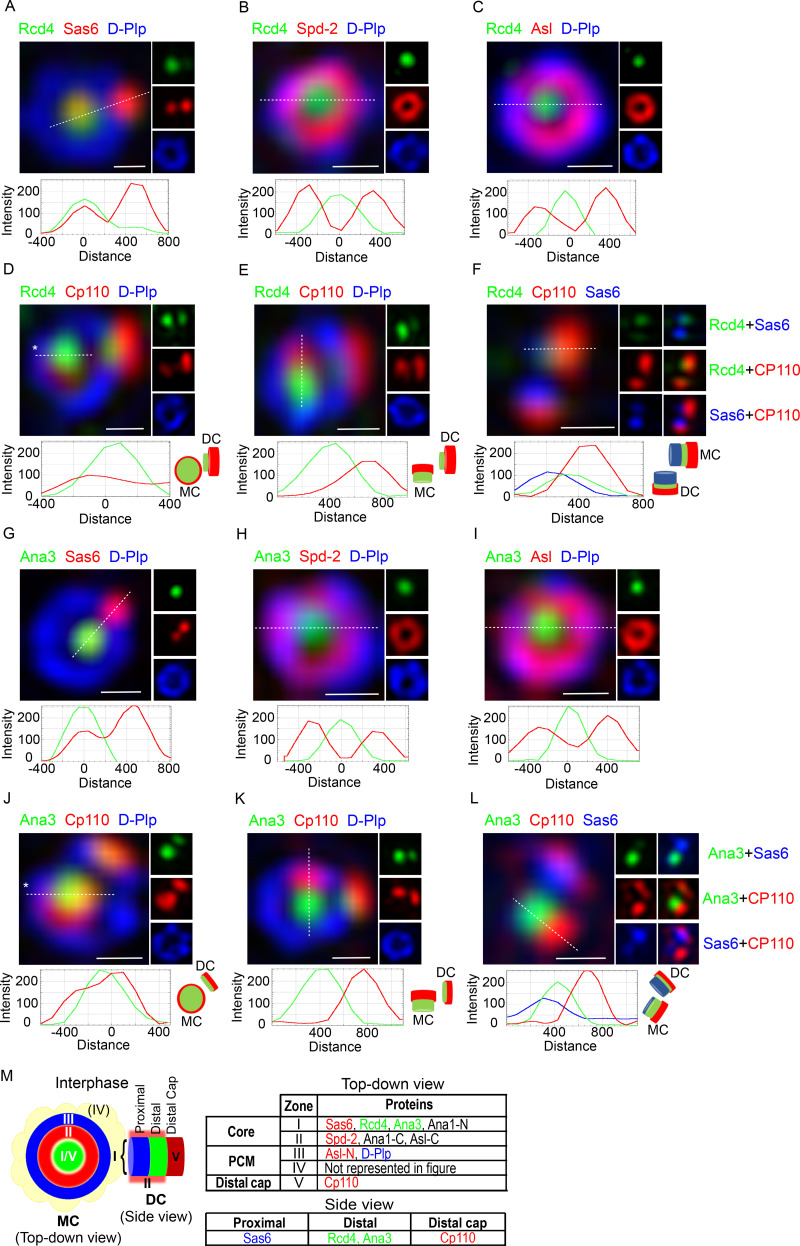
**Rcd4 and Ana3 localize to the centriole lumen above the cartwheel.** **(A–L)** 3D-SIM images of interphase D.Mel-2 cells expressing Rcd4-GFP and GFP-Ana3 (expressed under constitutive *actin-5C* promoter) relative to zone I marker Sas6 (A and G), zone II marker Spd-2 (B and H), zone III marker Asl (C and I), and zone V marker Cp110 (D and J). Centrioles imaged vertically to reveal Rcd4 (E) and Ana3 (K) localization relative to distal end marker Cp110. Side-profile localization of Rcd4 (F) and Ana3 (L) relative to centriole proximal and distal end markers, Sas6 and Cp110, respectively. D-Plp was used as a centriole marker. Cells were immuno-stained to reveal GFP, Sas-6, Spd-2, Asl, Cp110, and D-Plp. Fluorescence intensity along the dotted line drawn in each image is plotted as a function of the distance along the line from the core of the centriole or along the proximal-distal axis. Asterisks, titled centrioles. Scale bars, 200 nm. **(M)** Schematic of the zonal architecture of a centrosome showing a top-down view of the mother centriole (MC) and a side view of the daughter centriole (DC). The table indicates representative centriolar proteins for each zone, color-coded as demonstrated in panels A–L. N/C-Ana1 and Asl-C have been additionally included as examples of linearly arranged proteins spanning different zones. Zone IV (PCM) is not represented in this figure, which focuses only on interphase centrosomes.

Our above findings and previous evidence (Dobbelaere et al., 2008) indicate that Rcd4 is required for centriole duplication. To gain insight into its role in centriole duplication, we wished to determine its precise localization in the centriole and when it is recruited in the centriole duplication process. To address Rcd4’s localization, we applied super-resolution structured illumination microscopy (SIM) to cultured D.Mel-2 cells expressing GFP-tagged Rcd4. This revealed Rcd4 to localize in the core of the centriole (zone I), close to Sas6, which resides in the proximal interior part of the mother centriole and at the nascent procentriole in interphase cells (Fig. 5 A). Its presence within zone I was further indicated by its localization at the center of the ring of staining given by antibodies against spindle defective 2 (Spd2; Fig. 5 B), which in interphase D.Mel-2 cells colocalizes with Sas4 in zone II and at the center of the rings of Asl and D-Plp, which mark zone III (Fig. 5 C; Fu and Glover, 2012).

In the great majority of cases, the peak of Rcd4 staining intensity was positioned at the center of the rings of Spd2, Asl, or D-Plp staining. However, occasionally the staining pattern was displaced to one side (an example is given for costaining with Sas6 in Fig. 5 A). Such a pattern arises when two proteins lie on different planes on the proximal-distal axis of the centriole and when the centriole being observed is tilted. To determine Rcd4’s localization relative to the proximal and distal ends of the centriole, we also localized Rcd4 with respect to Cp110, which forms a cap on the distal end of the centriole (Fig. 5, D–F). In centrioles tilted from the vertical, we observed that the Rcd4 and Cp110 staining was not fully overlapping within the D-Plp ring (Fig. 5, D and E), and in centrioles that were displayed horizontally, Sas6 and Cp110 could be seen at the respective proximal and distal ends with Rcd4 between the two (Fig. 5 F). Thus, we conclude that Rcd4 lies within the lumen of the centriole (zone I), between Sas6 at the proximal end and the distal cap of Cp110.

We performed an analogous series of staining experiments to localize Ana3 with respect to the same centriolar marker proteins (Fig. 5, G–L). This revealed Ana3 to have a similar distribution to Rcd4. It localized close to Sas6 in zone I of the mother and the procentriole in interphase cells (Fig. 5 G) and was at the center of the rings of Spd2, Asl, and D-Plp staining (Fig. 5, H and I). Moreover, in centrioles that were tilted relative to the axis of view, Ana3 appeared displaced from the Cp110 distal cap (Fig. 5, J and K), indicating that the two molecules lay on a different plane. This was confirmed in centrioles viewed on a horizontal plane where Sas6 could be seen at the proximal end, Ana3 in a central position, and Cp110 as the distal cap (Fig. 5 L). Together these results suggest the Ana3:Rcd4 complex is localized to the lumen of the centriole distal to the Sas6 cartwheel protein (Fig. 5 M).

**Figure S5.**
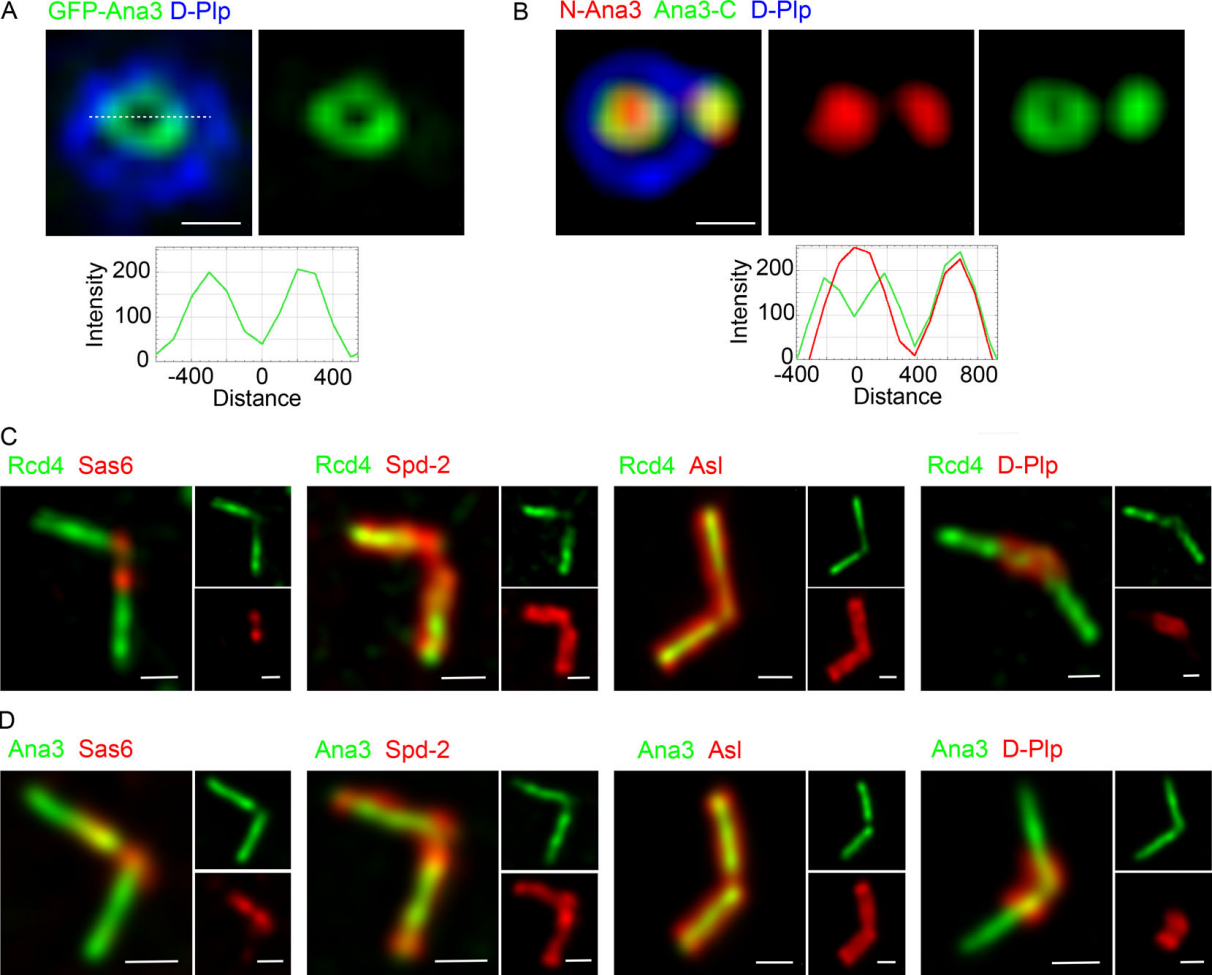
**The occasional ring-like distribution of Ana3 observed in cultured cells and the localisation of Rcd4 and Ana3 in the giant centrioles of primary spermatocytes. (A and B) Ana3 occupies a ring-like distribution in the centriole lumen. (A)** Occasionally observed 3D-SIM image of GFP-Ana3 occupying a ring-like distribution in interphase D.Mel-2 cells expressing GFP-Ana3 (under the control of constitutive *actin-5C* promoter) and immuno-stained to reveal GFP (green) and centriole marker D-Plp (blue). **(B)** Ana3 N- and C-terminal distribution; Ana3-GFP expressing D.Mel-2 cells (under the control of constitutive *actin-5C* promoter) were immuno-stained to reveal N-terminal Ana3 (using anti-Ana3 antibody generated against Ana3 1–275 aa), GFP (C-terminally tagged Ana3-GFP), and centriole marker D-Plp. Fluorescence intensity along the dotted line drawn in each image is plotted as a function of the distance along the line from the core of the centriole. Scale bars, 200 nm. **(C and D) **Rcd4 and Ana3 localization in the giant centrioles of primary spermatocytes. Giant centrioles of mature primary spermatocytes from flies expressing Ubq>GFP-Rcd4 (A)/Ubq>GFP-Ana3 (B; green) immuno-stained to reveal relative localizations to Sas6, Spd-2, Asl, and D-Plp (red). Scale bars, 0.5 µm.

Occasionally, N-terminally GFP-tagged Ana3 was observed to localize as a small ring (Fig. S5 A). Since it is a large protein of predicted molecular mass ∼225 kD, we wondered whether its N and C termini occupied distinct positions within zone I. To address this, we determined the relative positions of its C terminus, in a cell line expressing C-terminally GFP-tagged Ana3, and its N-terminal part using an antibody raised against an N-terminal, 1–275 amino acid fragment. As with GFP-Ana3, C-terminally GFP-tagged Ana3 also occasionally was revealed to have a small ring-like distribution within the D-Plp ring (Fig. S5 B). This indicates that Ana3 might potentially occupy a ring-like localization within the centriole lumen but is more often not visible, likely due to limitations in SIM resolution.

Although the above observations suggested that the Rcd4:Ana3 complex is located to the distal part of the centriole’s inner core, the short nature of centrioles in cultured *Drosophila* cells makes it difficult to distinguish this region from the proximal part. Therefore, to confirm the relative positions of Sas6 and Rcd4, we turned to the two pairs of elongated centrioles in fly primary spermatocytes and found that Sas6 (Persico et al., 2020) lay in the proximal part of zone I, while Rcd4 was prominent in the distal part (Fig. S5 C). We also found a similar spatial relationship between D-Plp (Fu and Glover, 2012) at the periphery of the proximal part of the centriole and Rcd4 within zone I of the distal part. Both Asl (Fu and Glover, 2012; Galletta et al., 2016) and Spd2 (Rodrigues-Martins et al., 2007a) extended further down the length of the centriole than Rcd4 (Fig. S5 C). Together, this confirmed our conclusion from cultured cells that Rcd4 occupies the more distal part of the lumen of the centriole. We found that Ana3 displayed a similar spatial relationship to these same centriole markers as Rcd4 (Fig. S5 D), supporting the presence of the two proteins in a complex in the distal lumen.

### Rcd4:Ana3 is recruited to daughter centrioles before their conversion to centrosomes

**Figure 6.**
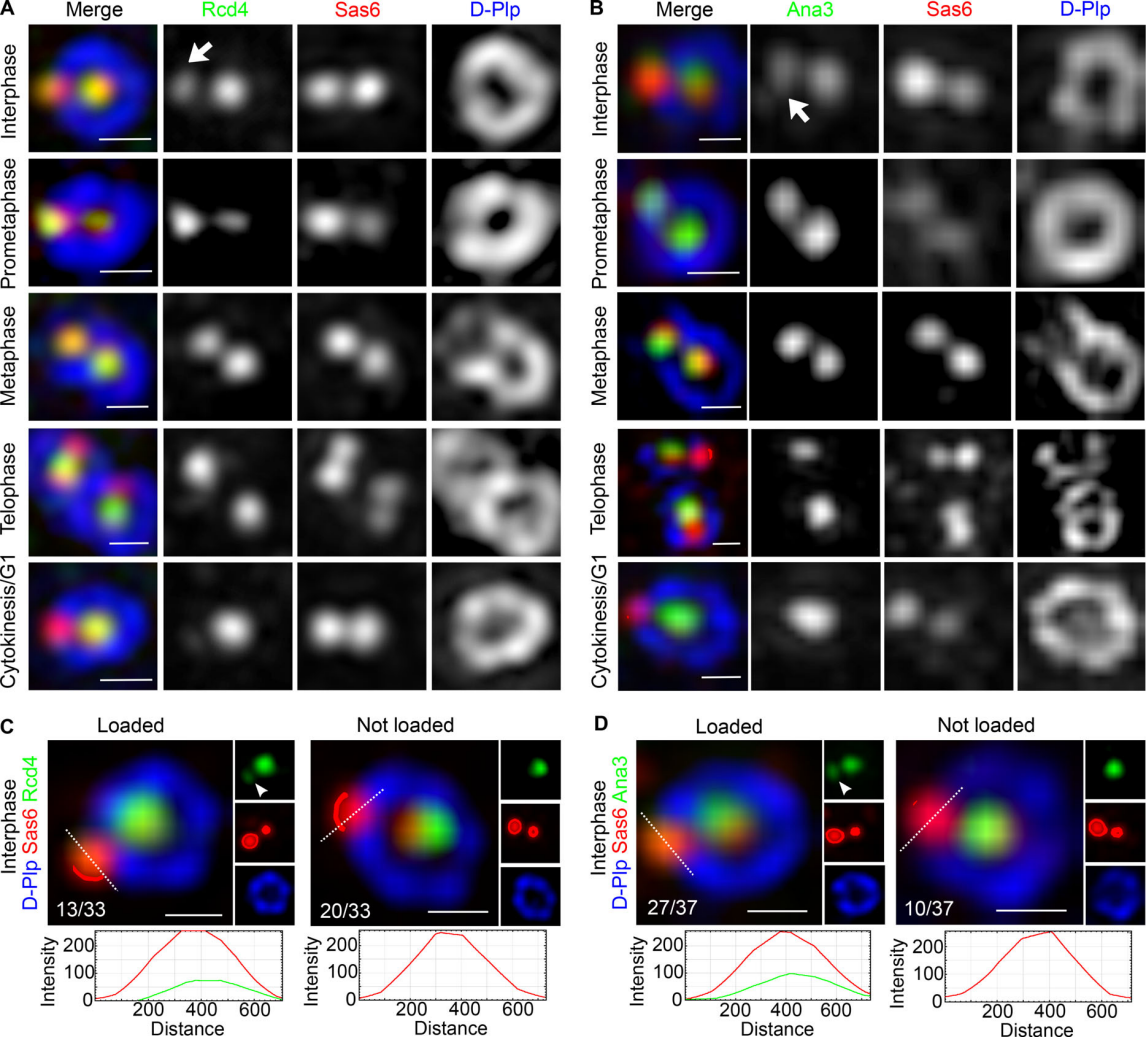
**Rcd4 and Ana3 are recruited to the procentriole in interphase. (A and B)** 3D-SIM images of Rcd4-GFP (A) and GFP-Ana3 (B; both expressed under the constitutive *actin-5C* promoter) throughout the D.Mel-2 cell cycle, relative to zone I marker Sas6 and zone III marker D-Plp. Cytokinesis/G1, cells in cytokinesis or already transitioned into G1 cell cycle phase. Arrows, the earliest stage of the cell cycle when Rcd4-GFP/GFP-Ana3 are recruited to the procentriole. **(C and D)** Proportions of interphase cells scored for loading of Rcd4-GFP (C) and GFP-Ana3 (D) onto the procentriole, relative to Sas6 and D-Plp. Rcd4-GFP, *n* = 33 and GFP-Ana3, *n* = 37 interphase cells were scored, N = two times. Cells were immuno-stained to reveal GFP (green), Sas-6 (red), and D-Plp (blue). Fluorescence intensity along the dotted line drawn in each image is plotted as a function of the distance along the line. Scale bars, 200 nm.

In cultured *Drosophila* cells, the major events of the centriole duplication cycle are tied to the passage through mitosis. Plk4 promotes the recruitment of first, Ana2 (Dzhindzhev et al., 2017), and then Sas6 (Dzhindzhev et al., 2014), to initiate procentriole formation immediately after mother and daughter centrioles disengage during anaphase/telophase. Interphase marks a period in which the new procentriole grows so that the cell enters mitosis with a full-length daughter centriole. As cells progress through prometaphase, Ana1 enables the recruitment of Asl, which will, following disengagement of mother and daughter, enable the association of pericentriolar material in the conserved process of centriole to centrosome conversion (Izquierdo et al., 2014; Wang et al., 2011; Fu et al., 2016). To assess when Rcd4 was loaded onto the procentriole, we followed its localization as cells progress through the division cycle (Fig. 6 A). Cells enter mitosis and progress through prometaphase with Sas6 and Rcd4 on both mother and daughter centriole. We define the transition between procentriole and daughter centriole to be at mitotic entry. At this time, daughters have an immature ring of D-Plp, which is completed as the centriole is converted into a centrosome in progression through mitosis. After mother and daughter centrioles disengage, Sas6 is recruited to start the formation of the new procentriole at late telophase. Rcd4 was not recruited onto the site of procentriole formation at this time and was also absent from this site during cytokinesis/G1 phase (Fig. 6 A). Thus, it appears that Rcd4 is recruited into zone I during interphase after the formation of the cartwheel at the end of mitosis.

We also performed a similar series of observations to determine when Rcd4’s binding partner, Ana3, was recruited. This revealed Ana3 to follow a similar temporal pattern to Rcd4; it was associated with the daughter centriole on mitotic entry and was not recruited alongside Sas6 following centriole disengagement (Fig. 6 B).

These observations raised the question of when during interphase Rcd4 and Ana3 are loaded onto the procentrioles. Unfortunately, we could not address this question directly as there are no good ways to synchronize cultured *Drosophila* cells. However, we noted that both Rcd4 and Ana3 were only loaded onto a fraction of interphase procentrioles and so argued that the proportion of “loaded procentrioles” would give an indication of their relative times of loading. Quantitation revealed that 39% (*n* = 33; Fig. 6 C) of interphase procentrioles were loaded with Rcd4 in comparison to 73% (*n* = 37; Fig. 6 D) that were loaded with Ana3. Thus, we conclude that Ana3 is likely loaded onto the procentriole ahead of Rcd4 during progression through interphase.

### Loading of Rcd4 requires Ana3 and is a prerequirement for centriole-centrosome conversion

**Figure 7.**
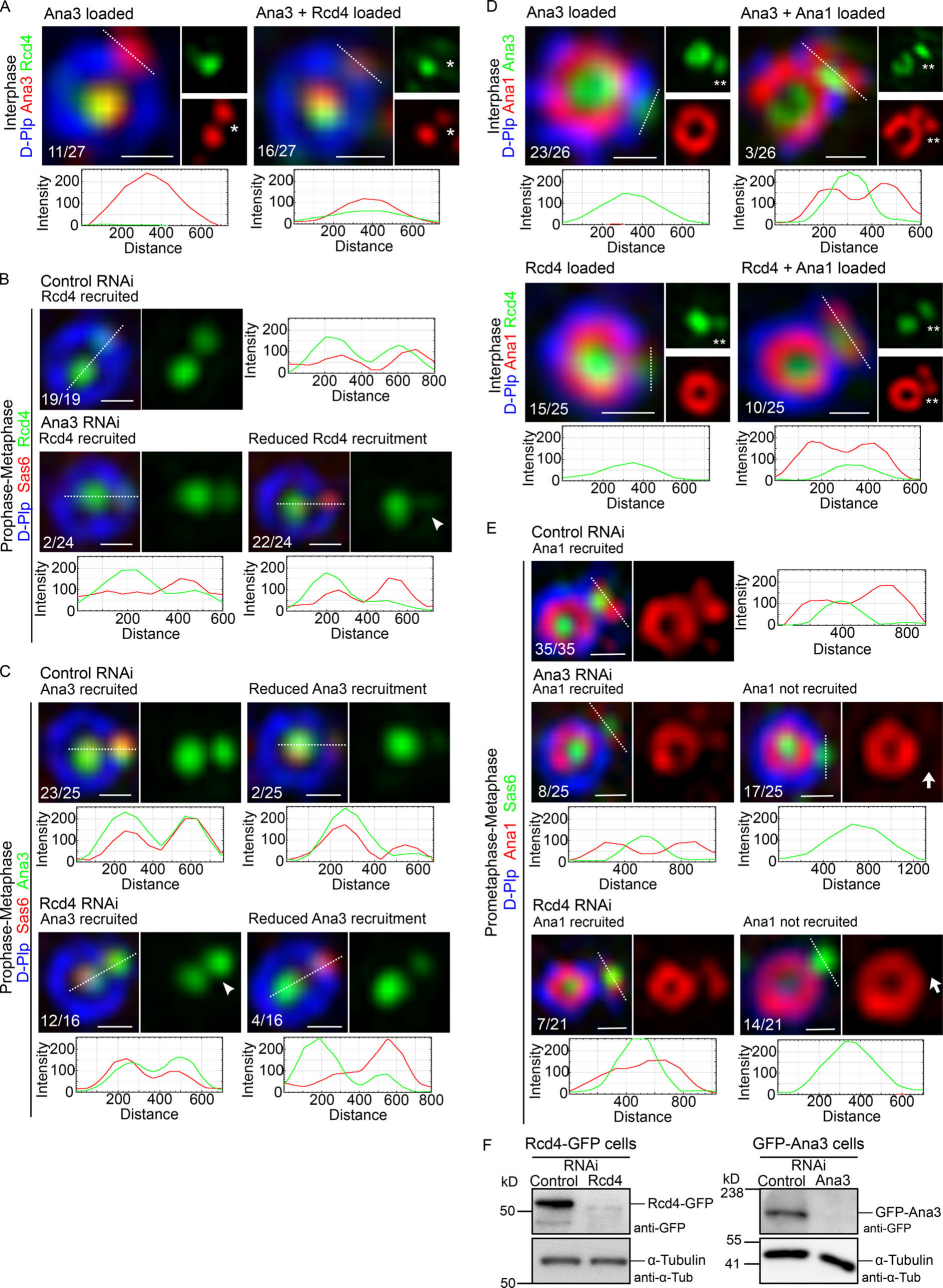
**Rcd4 recruitment to the daughter centriole is dependent on Ana3, while Ana1 recruitment requires prior loading of both Rcd4 and Ana3.** **(A)** Ana3 is loaded onto the daughter centriole ahead of Rcd4. 3D-SIM images of proportions of interphase D.Mel-2 cells expressing GFP-tagged Rcd4 (under constitutive *actin-5C* promoter) scored for loading of (i) only Ana3 or (ii) both Rcd4-GFP and Ana3 onto the daughter centriole. Cells were immuno-stained for GFP (green), Ana3 (red), and zone III marker D-Plp (blue), *n* = 27 centrosomes were scored, N = two times. Asterisk, loading of Rcd4-GFP/Ana3 at the daughter centriole. **(B)** Rcd4 recruitment to the daughter centriole is dependent on Ana3. D.Mel-2 cells expressing Rcd4-GFP (as in A) were treated with control and Ana3 dsRNA for 5 d. Mitotic cells (prophase-metaphase) were scored for recruitment of Rcd4-GFP to the daughter centriole. Cells were immuno-stained to reveal GFP (green) relative to zone I marker Sas6 (red) and zone III marker D-Plp (blue), *n* = 19 centrosomes scored in control dsRNA-treated cells and *n* = 24 centrosomes scored in Ana3 dsRNA-treated cells; N = at least three times. Arrowhead, diminished recruitment of Rcd4-GFP in Ana3 dsRNA-treated cells. **(C)** Recruitment of Ana3 to the daughter centriole is independent of Rcd4. D.Mels-2 cells expressing GFP-tagged Ana3 (under constitutive *actin-5C* promoter) were treated with control and Rcd4 dsRNA for 6 d. Cells were scored as in B for recruitment of GFP-Ana3. Cells were immuno-stained to reveal GFP (green), Sas6 (red), and D-Plp (blue), *n* = 25 centrosomes scored in control dsRNA-treated cells and *n* = 19 centrosomes scored in Rcd4 dsRNA-treated cells; N = at least three times. Arrowhead, recruitment of GFP-Ana3 to the daughter centriole in Rcd4 dsRNA-treated cells. **(D)** Ana1 is loaded onto the daughter centriole after Ana3 and Rcd4. Proportions of interphase D.Mel-2 cells expressing (i) GFP-tagged Rcd4 (as in A) and (ii) GFP-tagged Ana3 (as in C) scored for loading of only GFP-Ana3/Rcd4-GFP or both GFP-Ana3/Rcd4-GFP and Ana1 to the daughter centriole. Cells were immuno-stained to reveal GFP (green), Ana1 (red), and D-Plp (blue), *n* = 26 centrosomes were scored for Ana3-Ana1 recruitment and *n* = 25 centrosomes were scored for Rcd4-Ana1 recruitment; N = two times. Double asterisks, loading of GFP-Ana3/Rcd4-GFP and Ana1 at the daughter centriole. **(E)** Loading of Ana3 and Rcd4 is required for Ana1 recruitment. D.Mels-2 cells were treated with control dsRNA for 6 d, Ana3 dsRNA for 5 d, and Rcd4 dsRNA for 6 d. Mitotic cells (prometaphase-metaphase) were scored for recruitment of Ana1 to the daughter centriole. Cells were immuno-stained to reveal Ana1 (red) relative to Sas6 (green) and D-Plp (blue), *n* = 35 centrosomes scored in control dsRNA-treated cells, *n* = 25 centrosomes scored in Ana3 dsRNA-treated cells, and *n* = 21 centrosomes scored in Rcd4 dsRNA-treated cells; N = two times. Arrows, lack of Ana1 recruitment to the daughter centriole in Ana3 and Rcd4 dsRNA-treated cells. Fluorescence intensity along the dotted line drawn in each image is plotted as a function of the distance along the line. Scale bars, 200 nm. **(F)** Western blot showing efficiency of RNAi depletion. Cell lines expressing GFP-tagged Rcd4/Ana3 were treated with respective dsRNAs and analyzed by Western blotting against GFP as a measure of depletion efficiency. N = two times.

To confirm our conclusion about the order of loading and hence formation of the Rcd4:Ana3 complex, we fixed and stained D.Mel-2 cells expressing Rcd4-GFP so we could simultaneously reveal Ana3 (Fig. 7 A). Rcd4 was only present on procentrioles of interphase cells that were positive for Ana3. We found that 59% (*n* = 27) of centrioles in which Ana3 had been loaded were also positive for Rcd4. The presence of 30% (*n* = 27) Ana3-positive procentrioles that did not stain for Rcd4 in interphase indicates that Ana3 loads first as the procentriole is elongating before mitosis.

The above observation also inferred that loading of Rcd4 would be dependent upon Ana3. To test this, we depleted Ana3 and Rcd4 by RNAi in separate experiments and determined the consequences of the knockdown of one protein upon the recruitment of its partner. We scored the degree of recruitment that had been achieved by the prometaphase/metaphase stage as, in control cells, recruitment is completed by this time point. We found that depletion of Ana3 led to a failure of Rcd4 recruitment in 93% (*n* = 24) of procentrioles (Fig. 7 B). In comparison, Ana3 was still robustly loaded onto 75% (*n* = 16) of procentrioles following Rcd4 depletion and was weakly present on the remaining 25% (Fig. 7 C). Thus, the loading of Rcd4 is dependent upon the loading of Ana3 but not vice versa.

We then considered the consequences of a failure to load either Ana3 or Rcd4 in interphase upon the next major event in centriole duplication, centriole to centrosome conversion, that takes place as cells progress through mitosis. We chose to monitor the initiation of this process that we previously showed to be marked by the recruitment of Ana1 before mitotic entry to 19% (*n* = 90) of interphase procentrioles (Fu et al., 2016). In accord with this previous measurement, we found that when we examined the 73% of interphase procentrioles that had recruited Ana3, 12% (*n* = 26) of these had recruited Ana1 (Fig. 7 D). Similarly, when we examined the 39% of interphase procentrioles that had recruited Rcd4, 40% (*n* = 25) of these had recruited Ana1 (Fig. 7 D). Together, these findings are consistent with an order of recruitment of first Ana3 and then Rcd4 in interphase followed by Ana1 before the transition to mitosis.

We next assessed the requirement of the prior loading of Ana3 or Rcd4 for the recruitment of Ana1 and thereby, the onset of centriole to centrosome conversion. To this end, we assessed the formation of the nascent ring of Ana1 in prometaphase and metaphase cells following depletion of Ana3 or Rcd4 by RNAi (Fig. 7 E). Whereas Ana1 had been recruited onto the daughter centriole in 100% of control RNAi-treated mitotic cells, this was reduced to 32% (*n* = 25) in Ana3-depleted cells and 33% (*n* = 21) of Rcd4-depleted cells. Thus, the recruitment of Ana1 is severely compromised following depletion of either Ana3 or Rcd4. Consequently, centriole to centrosome conversion cannot be correctly initiated in these cells.

## Discussion

In this study, we have generated hypomorphic and amorphic mutations of the *Rcd4* gene in *Drosophila* that have allowed us to demonstrate its requirement for centriole duplication and the correct development of cilia in the neurosensory chordotonal organs. Basal bodies and cilia are completely absent in the chordotonal organs of the *rcd4^2^*-null flies that consequently show extreme loss of coordination. The partial loss of cilia in the *rcd4^1^* hypomorph suggests that centriole duplication was not completed during the final rounds of the division cycle in the scolopidium cell lineage of the femoral chordotonal organs. This conclusion gains support from the absence of centrobin staining of the basal bodies of *rcd4^1^* mutant scolopdia, indicating failure to produce daughter centrioles in the mitoses preceding differentiation. As a consequence, instead of the pair of cilia present in WT scolopidia, there was usually either a single cilium or none at all in *rcd4^1^* scolopidia. When present, the cilia showed defects in symmetry and structure extending along their length and often made abnormal associations with the ciliary membrane. These findings reveal a requirement for Rcd4 to generate basal bodies that are structurally able to template the formation of cilia and associate with cell membranes correctly.

In accord with the requirement for centriole duplication apparent from the *rcd4^1^* and *rcd4^2^* mutant phenotypes, we found that embryos derived from *rcd^1^* mothers fail in development as a result of mitotic defects associated with loss of centrosomes. We could not assess this directly with *rcd4^2^* females as they were too severely uncoordinated to be able to mate. However, following rescue of *Rcd4* function in the nervous system, it was possible to generate perfectly coordinated and motile females that were unable to produce viable offspring. Such females generated embryos in which failure of the centriole duplication cycle led to massive mitotic abnormalities in early syncytial embryos, accounting for the maternal effect lethality.

*rcd4^1^* encodes an N-terminally truncated protein comprising only the 68 C-terminal amino acids of the 199 amino acid protein. The hypomorphic nature of this *rcd4^1^* mutant allele indicates that there is some residual function in the approximate C-terminal third of the Rcd4 protein produced by this mutant. It is this C-terminal part of Rcd4 that shares the greatest homology with its human counterpart protein phosphatase 1 regulatory subunit 35 (PPP1R35), with which it shows overall 24% similarity. PPP1R35 is annotated as a protein phosphatase 1 (PP1) regulatory subunit and is reported to bind and inhibit PP1 (Hendrickx et al., 2009). However, it was not possible to identify any PP1 in proximity to PPP1R35 in BioID assays (Sydor et al., 2018), and the PP1 interacting motif was found not to be essential for centriole function in human cells (Fong et al., 2018). In a similar vein, we did not identify any PP1 isoform to coaffinity-purify with Rcd4. We did, however, identify Ana3 as its copurifying partner in extracts of cultured *Drosophila* cells. This accords with the identification of the Ana3 homologue, Rotatin, in proximity to PPP1R35 by BioID (Sydor et al., 2018). This, together with the coimmunoprecipitation and colocalization of Rotatin and PPP1R35 by 3D-SIM, led Sydor et al. (2018) to suggest that Rotatin and PPP1R35 form a complex. However, this was not supported by direct evidence of any physical interaction. Our demonstration that purified Rcd4 and Ana3 proteins can form complexes in vitro provides direct evidence for a physical interaction between the counterpart proteins in *Drosophila*. Moreover, Rcd4 specifically binds to the C-terminal half of Ana3, and it appears to interact more strongly with Ana3-C than with full-length Ana3. Although we do not yet know the underlying reason for this, we speculate that full-length Ana3 might adopt an inhibitory conformation that partly masks the Rcd4 binding region.

Our observations of centrioles in cultured *Drosophila* cells by 3D-SIM placed the Rcd4 protein as a component of the distal part of zone I. It is localized close to Sas6, which forms the molecular skeleton of the cartwheel that is assembled upon the initiation of procentriole formation. As the centrioles in cultured *Drosophila* cells are extremely short, we can say very little about the positioning of Rcd4 along the proximo-distal axis of the centriole other than that it lies in a domain between Sas6 at the proximal end and the distal cap formed by Cp110 and its interacting proteins. However, we could confirm its prominent localization in the distal part of zone I in the elongated centrioles of primary spermatocytes. This is in an analogous position to PPP1R35, which was described by Sydor et al. (2018) to lie in the proximal lumen of the centriole above the cartwheel.

Not only are the Sas6:Ana2 and Rcd4:Ana3 complexes localized in distinct parts of zone I, but whereas Sas6 and Ana2 are recruited upon procentriole formation immediately after procentriole disengagement during anaphase/telophase, Rcd4 and Ana3 are recruited at a later stage during interphase. Ana3 is the first to be recruited, and is present on 73% of interphase procentrioles, followed by Rcd4, which is present on 39% of procentrioles. Our study indicates not only that Ana3 is recruited to the procentriole ahead of Rcd4, but also that Rcd4 requires Ana3 to be able to load. In this respect, the loading dependency differs from human cells, where loading of the two corresponding proteins appears to be interdependent (Sydor et al., 2018). Whether this apparent discrepancy is due to functional differences of the homologous proteins between the different model systems remains to be elucidated.

**Figure 8.**
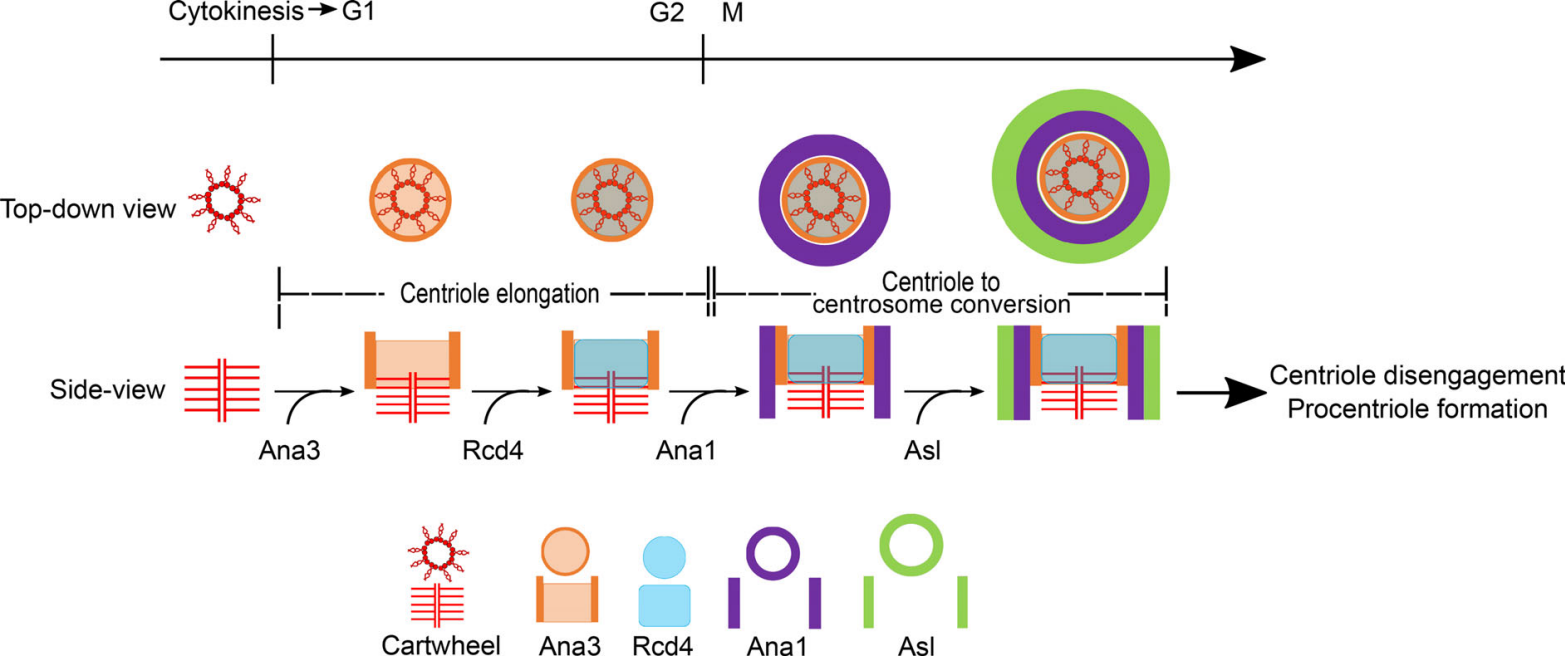
**Model illustrating recruitment of Ana3 and Rcd4 and their role in directing centriole-to-centrosome conversion of the assembling daughter centriole.** Both Ana3 and Rcd4 are recruited to the procentriole in interphase where Ana3 loading precedes Rcd4. Ana3 and Rcd4 are recruited to the centriolar lumen where Ana3 localizes as a small ring-like structure; both proteins occupy a region in between the proximal-distal ends of the centriole. Simultaneously, the assembling procentriole undergoes centriole elongation. Loading of both proteins then enables the recruitment of Ana1 to the assembling daughter centriole to direct centriole-to-centrosome conversion facilitated by the consequent recruitment of Asl (Fu et al., 2016).

The recruitment of Rcd4 takes place around the same time as Cep135, which is present on 30% of interphase procentrioles and which is required for subsequent recruitment of Ana1 (Fu et al., 2016). The recruitment of Ana1 ahead of mitotic entry marks the onset of the conversion of the daughter centriole to a centrosome during mitotic progression in both *Drosophila* and human cells (Izquierdo et al., 2014; Wang et al., 2011; Fu et al., 2016). Both Ana3 and Rcd4 are also required for Ana1 recruitment in *Drosophila* cells. This concurs with findings in human cells where Chen et al. (2017) have shown that loss of Rotatin, the human counterpart of Ana3, completely prevented loading of Ana1’s counterpart, Cep295, and consequently all of the centriole proteins that load late in the duplication cycle. Similar results were reported as a consequence of depletion of PPP1R35 (Fong et al., 2018; Sydor et al., 2018). Together, this suggests that the Rcd4:Ana3 heterodimeric complex may serve as a platform to recruit or anchor Ana1, through a role in setting up correct centriole structure to enable subsequent centriole to centrosome conversion in the mitoses of somatic cells rather than directly in conversion per se (Fig. 8).

Whereas one group have emphasized a requirement for PPP1R35 for centriole to centrosome conversion in human cells (Fong et al., 2018), another group emphasized the requirement for PPP1R35 in concert with Rotatin to regulate centriole length (Sydor et al., 2018). The short nature of centrioles in somatic *Drosophila* cells makes it difficult to assess the requirement of Rcd4 for centriole elongation. Therefore, we turned our focus to the giant centrioles of the fly primary spermatocytes. While the *rcd4*-null allele has some effect on the centriole duplication cycle of the male germ line cells, it does not affect the length of the remaining centrioles. This accounts for the fertility of *rcd4^2^* males and is a striking indication that, although essential in somatic tissues, the Rcd4 protein is not absolutely required for centriole or basal body formation in the male germ line. To assess whether Ana3 is required for male germ line development, we have used CRISPR/Cas9-mediated mutagenesis to generate an *ana3* null allele. Such *ana3* nulls are male sterile and display extremely few but highly aberrant centrioles in the male germ line (data not shown). These findings concur with a previous report (Stevens et al., 2009) and indicate that, in contrast to Rcd4, Ana3 is absolutely required to generate centrioles for spermatogenesis.

In conclusion, our combined findings point to a need for the coordinated recruitment of Ana3 and then Rcd4 to enable the later stages of centriole assembly, which are a precondition for centriole to centrosome conversion in somatic cells. Such failures are less extreme in *rcd4^1^* mutants that have enough residual function to generate fChOs with single maternal centriole-derived basal bodies sufficient to generate aberrant cilia, in contrast to the *rcd4^2^ null*, where no axonemal microtubules form in the fChOs. The disparity in requirements for Rcd4 in somatic tissues and in the male germ line is striking, and it will be a topic of future interest to determine why there are similar requirements for Ana3 and Rcd4 for development of procentrioles in somatic cells but different requirements in spermatogenesis.

## Materials and methods

### Fly stocks and husbandry

All fly stocks were maintained at 25°C, 60–80% humidity, and on standard cornmeal-yeast-sucrose media.

Embryos were collected on apple juice concentrate-agar-glucose plates at 25°C.

*w^[1118]^* flies were used as WT control flies.

The specific stocks used and fly lines generated in this study are listed in Table S1.

*rcd4* mutant fly lines were generated using a CRISPR/Cas9 mutagenesis tool for *Drosophila* genome engineering (Port et al., 2014). Targeting sequences for gRNAs were cloned into pCFD3 (gRNA-expressing vector, Addgene, 49410; Port et al., 2014) as described. For *rcd4^1^*, a pair of gRNA-expressing plasmids (gRNA-1 and 1′ as in Fig. 1 A; targeting the 5′ and 3′ ends of *Rcd4*) were directly injected into nos-Cas9 embryos (Bloomington, 54591) by BestGene Inc. For *rcd4^2^*, a second pair of gRNA-expressing plasmids (gRNA-2 and 2′ as in Fig. 1 A) were used to generate two transgenic fly lines (gRNA-2: integrated on the second chromosomal attP40 landing site; gRNA-2’: integrated on the third chromosomal attP2 landing site) by site-specific recombination via φC31 integrase-mediated cassette exchange (Groth et al., 2004; Bischof et al., 2007) by BestGene Inc. Both transgenic flies were then crossed to achieve a double gRNA expressing fly line (targeting 5′ and 3′ ends of *Rcd4*). gRNA-mediated double-strand breaks were induced by crossing the transgenic double gRNA expressing flies to nos-Cas9. The emergent G0 flies (cut starters) were individually crossed to second chromosome balancer lines, and candidate stocks were established and screened for deletion by PCR. Precise breakpoints of two mutant alleles, *rcd4^1^* (NT_033779.5:g.8382966_8383410del) and *rcd4^2^* (NT_033779.5:g. 8382933_8383546 del), were determined by Sanger sequencing. Primers used are described in Table S2.

Transgenic *Rcd4* fly lines for rescue experiments and localization studies were generated by integrating all transgenes via φC31 integrase-mediated cassette exchange into the same *attP40* landing site for standardized expression levels (University of Cambridge Department of Genetics Fly Facility). Gateway system (Thermo Fisher Scientific)–compatible *Rcd4* entry clones were generated by amplifying the coding sequence from SD16838 cDNA clone (Drosophila Genomics Resource Center) and cloning into a pDONR221 entry vector (Invitrogen). The entry clones were recombined into pPGWattB, pUGWattB, pUWGattB *Drosophila* destination vectors (Kovacs et al., 2018). Transgenic *ana3* fly lines for localization studies were similarly generated by integrating the transgene into the attP2 landing site (see Ana3 CDS synthesis).

### Negative geotaxis experiment (coordination test)

To measure locomotor coordination, WT, *rcd4* mutant, and rescued flies were raised until pupal stage. Pupae were gently transferred into fresh vials, which were placed on their sides to prevent uncoordinated flies from sticking to the media upon eclosion. Cohorts of 10 adult flies (five males and females each, aged 2 d) were transferred into fresh vials 1 d before the experiment. Just before the test, flies were transferred without anesthesia into clear, empty testing vials. The vials were illuminated from above. Flies were gently tapped down to the bottom of the vial and then given 30 s to climb up. The number of flies that crossed the 5-cm mark were then recorded and statistically analyzed. The coordination test was repeated three times for each cohort. Three independent cohorts of 10 flies were tested per genotype.

### Fertility test

The coordination defect of *rcd4* mutants was rescued with a pan-neural driver *elav-GAL4* and WT transgene *UAS-GFP-Rcd4* before fertility test to ensure coordination defects did not interfere with mating ability. To test female fertility, WT, neuronally rescued *rcd4* mutants and rescued777 virgin females (aged 4 d) were collected and individually mated with two WT males (aged 2–4 d). The crosses were kept at 25°C for 6 d, after which the adults were removed. The number of eclosed progeny in each vial was recorded and statistically analyzed. Male fertility was similarly tested as described; individual males were mated with two WT virgin females (aged 4 d before mating to test for virginity). 10 crosses per genotype were evaluated in two independent experiments. Vials in which any adults died were excluded from the analysis.

### Immunostaining and confocal microscopy of fly tissues

#### Embryos

For immunostaining of *Drosophila* embryos, 0–2 h synchronized embryos were dechorionated in 50% bleach for 2.5 min, devitellinized in a 1:1 ratio of methanol and heptane, fixed, and stored in methanol. Before immunostaining, the embryos were rehydrated by successive washes in methanol/PBS (75–50%-25% methanol in PBS) for 10 min at each methanol dilution followed by a final 15-min wash in PBS. The embryos were then blocked in PBS/0.1% Triton X-100 (PBST) containing 10% FBS (Gibco) for 1 h followed by overnight incubation at 4°C with the following primary antibodies: mouse anti-α-tubulin (1:300, clone DM1A, Sigma-Aldrich, T6199-200UL) and rabbit anti-Asl (1:500, developed in the laboratory at the Department of Genetics, University of Cambridge, UK; Dzhindzhev et al., 2010). After three 20-min PBST washes, the appropriate secondary antibodies were added for 4 h at 25°C. Following a final round of three 20-min PBST washes, the embryos were mounted in Vectashield containing DAPI (Vector Laboratories).

#### Testes

Testes were dissected from pharate adults in PBS and transferred to a drop of 5%-glycerol/PBS on a microscope slide. The testes were then squashed with a coverslip, snap-frozen in liquid nitrogen, and fixed in chilled methanol for 3 min. The samples were rehydrated in PBS for 5 min, washed in PBST for 10 min, and incubated overnight in a humid chamber at 4°C with the following primary antibodies: rat anti-Sas6 (1:200, developed in the laboratory; Dzhindzhev et al., 2014), rabbit anti-Spd2 (1:400, developed in the laboratory; Fu and Glover, 2012), rabbit anti-Asl (1:6,000, developed in the laboratory; Dzhindzhev et al., 2010), and chicken anti-D-Plp (1:1,000, developed in the laboratory; Rodrigues-Martins et al., 2007b). The slides were washed thrice in PBST for 10 min and incubated with appropriate secondary antibodies in a humid chamber for 1 h at 25°C, followed by three 10-min washes in PBST and mounting in Vectashield containing DAPI.

#### Femoral chordotonal organs

For femoral chordotonal organ immunostaining, flies were raised until pharate adult stage. The pupal case was removed, and the whole pharate adult was fixed in 4% formaldehyde for 30 min, followed by three 10-min washes in PBST. The first pair of legs was finely dissected in PBS up until the region containing the chordotonal organs (Fig. 2 A), where the cuticle was opened to facilitate antibody diffusion. The leg fragments were then immuno-stained as described above for embryos, with primary antibody chicken anti-D-Plp (1:500). Alexa Fluor 647 phalloidin (1:100, Thermo Fisher Scientific, A22287) was added during secondary antibody incubation. The samples were mounted in Vectashield without DAPI.

Secondary antibodies conjugated with Alexa Fluor 488, 568, 594, and 647 (Life Technologies) were used at 1:500 dilution in each of the experiments described above.

#### Confocal microscopy

Microscopic images were collected on a Leica SP8 laser scanning confocal microscope (using Application Suite X software, LAS-X, Leica) using 40× (oil), 63× (oil), or 100× (oil) objectives, illuminated with a range of lasers from 405–639 nm and detected by Hybrid (avalanche photodiode/photomultiplier tube) or photomultiplier tube detectors. Images of giant centrioles from fly testes were deconvolved using Huygens Professional software. All processing and analysis of microscope images were performed with ImageJ (ImageJ 1.52p, National Institutes of Health).

### Centriole length measurements in spermatocytes

To measure centriole length in mature primary spermatocytes, only paired centrioles oriented perpendicular to the imaging axis were considered to avoid ambiguity in measurements contributed by the tilted conformation. Z-stacks of 0.5-µm steps, spanning the entire centriole volume, were taken on the Leica SP8 laser scanning confocal microscope, as described above. The length of the centrioles was measured using the line profile tool in ImageJ and was statistically analyzed.

### Transmission electron microscopy

Antennae of WT, *rcd4^1^*, and *rcd4^2^* mutants were dissected in PBS from mid-age pupae and transferred into 2.5% glutaraldehyde buffered in PBS overnight at 4°C. After prefixation, samples were washed in PBS and post-fixed in 1% osmium tetroxide in PBS for 2 h at 4°C. The materials rinsed in PBS were dehydrated in a graded series of ethanol (50%, 75%, 95%, 100%), embedded in a mixture of epon-araldite resin, and then polymerized at 60°C for 48 h. Ultrathin sections (50–60 nm thick) were obtained with an Ultracut E ultramicrotome (Reichert) equipped with a diamond knife, collected on formvar-coated copper grids, and stained with uranyl acetate and lead citrate. Images were acquired with a Tecnai Spirit Transmission Electron Microscope (FEI) operating at 100 kV and equipped with a Veleta CCD camera (Olympus).

### GFP-Trap affinity purification from D.Mel-2 cells for mass spectrometry

GFP-tagged Rcd4/Ana3 D.Mel-2 cells were harvested in large scale, pelleted down, and washed in PBS before being homogenized in ice-cold extraction buffer (75 mM Na-Hepes, pH 7.5, 150 mM NaCl, 2 mM MgCl_2_, 2 mM EGTA, 0.1% NP-40, 5 mM DTT, 5% glycerol, complete protease inhibitors, EDTA-free [Roche], and PMSF). The cell lysate was then centrifuged (12,000 rpm, 4°C, 20 min), and the supernatant was subjected to GFP-Trap purification (magnetic agarose beads, ChromoTek) as per the manufacturer’s instructions. The proteins were sent on beads to the Mass Spectrometry Laboratory, Institute of Biochemistry and Biophysics for mass spectrometry analysis. The bait proteins were digested with trypsin, and the resulting peptide samples were analyzed using an Orbitrap-LTQ mass spectrometer (Thermo Fisher Scientific). Acquired data were searched using the Mascot program (Matrix Science) against the *Drosophila melanogaster* database.

### Ana3 CDS synthesis

Due to repeated unsuccessful attempts in cloning full-length *Ana3* coding sequence, we used GeneArt Gene Synthesis (Thermo Fisher Scientific) to chemically synthesize the sequence. The *Ana3* coding sequence template used for gene synthesis was as reported by Stevens et al. (2009) except for a modification as detailed below, aimed to scramble the nucleotide sequence in a region suspected to interfere with previous unsuccessful cloning attempts. In the original Ana3-RA-CDS as reported in FlyBase, the nucleotide sequence starting at position 5221 is 5′-CTA​ATG​GCT​CGC​GTC​GCT​GAC​TTC​GAG​ACC​ACC​AAG​AAG​GAG​ATT​CCA​AAC-3′. The corresponding amino acid sequence starting at position 1741 is Leu Met Ala Arg Val Ala Asp Phe Glu Thr Thr Lys Lys Glu Ile Pro Asn. For the scrambled sequence in the template *ana3* coding sequence used for gene synthesis, the nucleotide sequence at position 5221 was instead 5′-**T**T**G**ATG​GC**CA**G**G**GT**G**GC**C**GA**T**TT**T**GA**A**AC**G**AC**G**AA**A**AA**A**GA**A**AT**C**CC**C**AA**T**-3′. However, the corresponding amino acid sequence starting at position 1741 is maintained as Leu Met Ala Arg Val Ala Asp Phe Glu Thr Thr Lys Lys Glu Ile Pro Asn. The alternative nucleotides used in the scrambled sequence are bolded, while integrity of amino acid sequence is maintained as in WT.

The *Ana3* coding sequence was assembled from synthetic oligonucleotides and/or PCR products and inserted into the pMS-RQ (spectinomycin) vector backbone (GeneArt Gene Synthesis service). The synthetic *Ana3* coding sequence was then amplified by PCR and cloned directly into Gateway entry vector pENTR1A (Invitrogen, 11813–011) by In-Fusion cloning (Takara) for downstream Gateway-compatible applications. Primers used are described in Table S2.

### Recombinant protein expression and in vitro binding assay

Gateway-compatible *Rcd4* and *Ana3* entry clones encoding full-length coding sequence (as described earlier) or fragments of Ana3 (as described in Fig. 4 A) were recombined with the following destination vectors: pDEST15 (for N-terminal GST fusion in *E. coli*; Invitrogen, 11802014) and pKM596 (for N-terminal MBP fusion in *E. coli*; Addgene, 8837; Fox et al., 2003) to generate expression constructs. The recombinant proteins (MBP, MBP-tagged Ana3 fragments, MBP-tagged Rcd4, GST, and GST-tagged Rcd4) were expressed in the *E. coli* strain Rosetta BL21(DE3) (Life Technologies) with 0.5 mM IPTG induction overnight at 18/20°C. Cells were lysed by sonication in ice-cold lysis buffer (20 mM Tris, pH 7.5, 200 mM NaCl, 1 mM dithiothreitol, 10% glycerol) supplemented with EDTA-free complete protease inhibitor cocktail (Roche) and 0.2 mg/ml lysozyme (Sigma-Aldrich). Primers used are described in Table S2.

In vitro binding assays were performed by incubating the cell lysate containing the bait protein on glutathione-sepharose 4B beads (GE Healthcare). After 1 h of rotation at 4°C, the beads were washed three times for 10 min with wash buffer (20 mM Tris pH 7.5, 200 mM NaCl, 1 mM DTT, 10% glycerol). Next, the MBP-tagged prey protein was added and incubated for 1 h at 4°C, followed by three 10-min washes with washing buffer. The proteins were eluted with Laemmli sample buffer and analyzed by SDS-PAGE with PageBlue protein staining solution (Thermo Fisher Scientific, 24620). For proteins at lower concentrations undetectable with PageBlue stain, Western blot analysis was conducted using primary antibody rabbit anti-MBP (1:5,000) and secondary antibody conjugated with HRP (1:10,000; Jackson ImmunoResearch).

### *S. cerevisiae* in vivo binding assay

Interaction between 6xHis-Rcd4 and GST-Ana3 was explored in *S. cerevisiae* using a modified method (Geymonat et al., 2007). Briefly, 6xHis-Rcd4 was expressed using plasmid pMH940 and GST-Ana3 using pMG1. All expression plasmids were obtained by recombination using strains MGY140 (for Ana3 constructs) and MGY139 (Mat a, ade5, ura3-5, trp1-289, his3, leu2, lys2Δ0, mob1::kanMX4, cdc28::LEU2, pep4::LYS2/YCplac33-MOB1-CDC28; for Rcd4 construct). After recombination, strains were mated and passed on fluoroorotic acid plates to obtain diploid strains stably expressing pair of Rcd4/Ana3 proteins. Strains were cultivated for 6 h in Yeast extract-Peptone-Galactose to induce expression of the recombinant proteins. Cell pellets were resuspended in Breaking Buffer (50 mM Tris, pH 7.5, 250 mM NaCl, 10% glycerol, 0.2% NP-40, 5 mM EDTA, 5 mM DTT, protease inhibitor cocktail, and 1 mM PMSF), and crude extract was obtained by vortexing in the presence of glass beads. GST-Ana3 constructs were purified on glutathione beads, washed four times with Washing Buffer (50 mM Tris, pH 7.5, 250 mM NaCl, 0.2% NP-40, and 5 mM DTT), and eluted with 20 mM reduced glutathione. The presence of 6xHis-Rcd4 bound to Ana3 was revealed by Western blotting using an anti-6xHis antibody.

GST-Ana3 full-length was similarly expressed and purified as described above for the in vitro binding assay with MBP-Rcd4 (Fig. S4 B).

Primers used are described in Table S2.

### Antibody generation

An Ana3 polyclonal antibody was generated against the N-terminal part of the protein (1–275 aa). MBP-tagged Ana3 fragment was expressed in *E. coli* strain Rosetta BL21(DE3) with 0.5 mM IPTG induction overnight at 20°C. The recombinant protein was purified on amylose resin (New England Biolabs) and eluted with maltose-elution buffer (20 mM Tris, pH 7.5, 200 mM NaCl, and 5% glycerol). The MBP-Ana3 fragment was then used as an antigen to immunize a rabbit by Moravian–Biotechnology Limited according to standard protocols. Specificity of the antibody was tested by immunostaining of WT and Ana3-RNAi D.Mel-2 cells.

### Cell culture, transfection, and RNAi

D.Mel-2 cells (Life Technologies) were grown at 25°C in Express Five SFM (Life Technologies) supplemented with L-glutamine (2 mM; Gibco) and penicillin–streptomycin (50 units/ml, 50 μg/ml; Gibco).

To establish GFP-tagged Rcd4 and Ana3 stable cell lines, Rcd4 and Ana3 Gateway entry clones were recombined with the following destination vectors: pAGW and pAWG (for Actin5C promoter-driven N- or C-terminal GFP fusion in *Drosophila* cells; Drosophila Genomics Resource Center). D.Mel-2 cells were then transfected with the plasmids using FuGENE-HD reagent (Promega) according to the manufacturer’s guidelines. 48 h post-transfection, 20 µg/ml of blasticidin (Melford Biolaboratories Ltd., B12200-0.1) was added to the media. The cells were maintained with blasticidin selection thereafter and passaged until a healthy, stable cell line was established (verified by Western blotting and immunostaining).

For GST (control), Rcd4 and Ana3 RNAi in D.Mel-2 cells, double-stranded RNAs (dsRNA) directed against the coding sequence were synthesized from PCR products of template coding sequence using the Megascript T7 kit (Ambion). The cells were transfected with dsRNA using TransFast Transfection Reagent (Promega) as per the manufacturer’s instructions and harvested for fixing after 5 d (Ana3 RNAi)/6 d (Rcd4 RNAi). RNAi depletion efficiency was accessed by treating GFP-tagged Rcd4/Ana3 cell lines with the respective dsRNAs (as described above), harvesting a total of 1.5 million cells, which were subjected to protein extraction in a 1:1 ratio of PBS with 2% benzonase (Merck Millipore) and Laemmli sample buffer. Western blot analysis was performed using primary antibodies: mouse anti-GFP (1:50; Roche, 11814460001) and mouse anti-α-tubulin (1:5,000) and respective secondary antibodies conjugated with HRP (1:10,000; Jackson ImmunoResearch).

### Structured illumination microscopy and data processing

For immunostaining of D.Mel-2 cells, cells were grown on concanavalin A–coated (Sigma-Aldrich) coverslips (no. 1.5, 0.17-mm thickness, Zeiss) for 2–4 h, fixed in chilled methanol for 3 min, rehydrated in PBS, and rinsed in PBST for 5 min. The cells were blocked in PBST containing 10% FBS for 30 min and incubated overnight at 4°C with the following primary antibodies: mouse anti-GFP (1:500), rat anti-Sas6 (1:500), rabbit anti-Spd2 (1:500), rabbit anti-Asl (1:6,000), rabbit anti-Cp110 (1:1,000, developed in the laboratory; Delgehyr et al., 2012), chicken anti-D-Plp (1:1,000), rabbit anti-Ana3 (1:1,000, developed in this study), rabbit anti-Ana1 (1:5,000, developed in the laboratory; Fu et al., 2016), and rabbit anti-phospho-histone H3 Ser10 (1:2,000; Millipore). The cells were washed for 5 min in PBST thrice before incubation for 1 h at 25°C with appropriate secondary antibodies. After a final round of three 10-min washes in PBST, the coverslips were mounted onto microscope slides with Vectashield with or without DAPI.

Secondary antibodies conjugated with Alexa Fluor 488, 568, and 594 (Life Technologies) were diluted 1:500, and DyLight 405 (Jackson ImmunoResearch) was diluted 1:100.

Super-resolution structured illumination microscopy images were acquired on a Deltavision OMX, 3D-SIM System, V3 BLAZE from Applied Precision (GE Healthcare) equipped with three sCMOS cameras, 405, 488, 592.5 nm diode laser illumination, with a 63x/1.4 NA oil Olympus objective, and standard excitation and emission filter sets. Image acquisition (512 × 512 pixels per inch) of each channel was done sequentially using three angles and five phase shifts of the illumination pattern. ZEISS immersion oils 1.512, 1.513, or 1.514 were applied to obtain the best resolution and to minimize spherical aberrations. Sections were acquired at 0.125 µm z steps.

Raw OMX data were reconstructed and channel-registered in SoftWoRx software version 5.5 (Applied Precision, GE Healthcare). Reconstructions were performed using channel-specific optical transfer functions, a Wiener filter of 0.002, and channel-specific K0 angles. Channel registration was done using the Image Registration parameters generated within the SoftWoRx software. Images were further processed in the software to obtain maximum intensity projections. Cropping of images was performed in ImageJ, and fluorescence intensity plots were generated using the RGB profiler plugin in ImageJ.

### Statistical analysis

The sample size (*n*), mean, SEM, and number of times each experiment was repeated (N) are indicated in corresponding figure legends. Data collected from coordination assay, fertility test, and centriole length measurements were analyzed with a two-tailed, unpaired *t* test by GraphPad Prism (version 5.01). P values for each analysis are indicated in corresponding figures; a 99% confidence interval was applied in all statistical tests.

### Online supplemental material

Fig. S1 shows that despite centriole loss in primary spermatocytes of *rcd4^2^* males, centriole elongation still proceeds normally in the remaining centrioles. Fig. S2 shows disrupted D-Plp staining in fChOs of *rcd4-*mutants, indicating defective basal bodies. Fig. S3 shows ciliary axoneme abnormalities observed in *rcd4^1^* fChOs. Fig. S4 shows binding between full-length Ana3 and Rcd4 in vitro and in vivo (*S. cerevisiae*) binding assays. Fig. S5 shows the occasional ring-like distribution of Ana3 observed in cultured cells and the localization of Rcd4 and Ana3 in the giant centrioles of primary spermatocytes. Table S1 lists the specific fly stocks used and generated in this study. Table S2 lists all the primer sequences used in this study.

## Supplementary Material

Table S1 shows fly stocks.

Table S2 shows oligo sequences.
